# It is not love, but social reproduction labor: nursing care in light of Nancy Fraser

**DOI:** 10.1590/1980-220X-REEUSP-2026-0127en

**Published:** 2026-09-28

**Authors:** Maria Raquel Gomes Maia Pires, Gabriela Duarte Almeida Mundim

**Affiliations:** 1 Universidade de Brasília, Faculdade de Ciências da Saúde, Departamento de Enfermagem, Brasília, DF, Brazil.; 2 Universidade de Brasília, Centro de Estudos Avançados Multidisciplinares, Brasília, DF, Brazil.

**Keywords:** Gender Stereotyping, Nursing, Nursing Care, Address, Feminism, Work

## Abstract

This is a theoretical study that adopts Nancy Fraser’s conception of care as social reproduction labor as a critique of the scientific self-discourse that defines care as “love” in nursing. We argue that care as social reproduction labor–purposeful labor aimed at meeting the social needs of the labor force in capitalist societies–reveals the weaknesses of the notion of “love” in nursing by exposing the material, intersectional, capitalist, and sociohistorical conditions that sustain it, thereby supporting counter-hegemonic practices. First, we reflect on the contradiction between care and capitalism. Subsequently, we undertake an immanent critique of the nursing self-discourse of love through the negative trihedron of care. Finally, we recalculate alternatives for a critical pathway regarding care work in the profession. We propose incorporating a gender perspective and dialogue with feminist epistemology into undergraduate and graduate curricula, with repercussions for scientific production and the political practice of nursing. The science we produce should problematize the social and gender contradictions within nursing, whose labor has been rigidly domesticated.

## INTRODUCTION

The concept of care in the scientific discourse of nursing–whether in education, practice, or professional organizations–has historically been conflated with duty or a moral attribute, grounded in mistaken notions of kindness, love, altruism, and related forms of benevolence, which supposedly elevate the nobility of the profession. There is a self-discourse among nurse researchers that positions care as a “loving practice”, “compassion”, and “the essence and disciplinary core” of the profession, at times treating these expressions as interchangeable, as though care and nursing were synonymous^(1,2,3,4)^. More explicitly, nursing theorists invoke “love and care explicitly, seeking their fusion into an expanded paradigm connected with the spiritual dimensions and vital processes of human experiences”^(4:132)^.

In contrast to this theoretical perspective, which presents itself as representative of “[...] Nightingale’s legacy and foundations”^(4)^, we conceive of care within the materiality of the social relations of production, i.e., as social reproduction labor according to Nancy Fraser. This labor is understood as a set of purposeful activities aimed at meeting the social needs of the labor force (food, shelter, clean housing, rest, well-being, health, education, among others) in capitalist societies. Reproductive labor is marked by intersectional conditions that exploit, dispossess, and domesticate (discipline) women’s labor unjustly, including nursing, as well as the labor of others who perform domestic tasks and care for children, older adults, and related dependents^(5,6)^. Understanding care as social reproduction labor under capitalism is not limited to the caregiving dimension of nursing but encompasses all activities performed by nurses and nursing technicians within public health and education policies (management, education, research, and care delivery), broadly understood as part of the material and symbolic reproduction of the labor force under capitalism. In this study, we use this conception of care to criticize the profession’s illusory discourse, namely, the discourse that conceives of care as “love” and related descriptors, briefly introduced above and further problematized in the following paragraphs.

First, in line with feminist epistemology’s reflections on the intersectional violence of gender, race, class, sexuality, and ageism against women–whether nurses or not–we question whether all people who perform care work are (or need to be) nurses, as nursing theorists seem to suggest^(1,2,3,4)^. Let us reflect together: what does the profession gain by heroically claiming, as a controversial and immutable “essence”^(1,4)^, responsibility for all the symbolic, institutional, and structural violence involved in the unequal assignment of care work to women? Would it not be more politically strategic to do the opposite, i.e., to confront internally the oppressive fallacy of statements that merely domesticate women’s bodies and obscure critical thinking in nursing? Certainly, this is only one of many questions, but much more is at stake when we hegemonically reproduce the ideological discourse of “love” in nursing. Let us explain further.

The discourse of the supposed “loving kindness” of care prevents the unveiling and collective confrontation of the contradictions embedded in the profession, such as: i - the epistemic fragilities surrounding care and the unsustainable efforts to transform it into a “moral ideal” or a “unitary science” of “infinite love” immune to criticism^(1,2,3,4)^; ii - the harmful influence of whiteness and the eugenic, racist, and classist origins of nursing professionalization, which generate discursive practices that internally reinforce oppression against women^(7,8,9)^; iii - the overexploitation of nursing labor within the capitalist context, low wages, and the widespread illness affecting nurses and the nursing workforce, often naturalized as professional duty, altruism, or supposed “heroism”^(10,11,12,13,14)^; iv - the symbolic violence of stereotyped and moralizing scientific discourses, uncritically perpetuated in professional education and the political practice of the profession^(15)^; v - the mistaken view that “society” stigmatizes and devalues nursing, as though nursing itself neither belonged to society nor contributed to how the profession is portrayed^(16)^.

A close look at the influence of the “Caritas process”^(1,4)^ in nursing clearly shows that society perceives us exactly as we insist on presenting ourselves (or denying ourselves), because we are part of that very society. We refer here to the comfortable argument that society devalues nursing and imposes gender stereotypes on the profession, as though we existed outside it^(14,16)^. However, if the prevailing and contradictory discourse embraced by many nurse scientists insists that we are morally superior beings, owners of care as the profession’s “unitary” essence, altruistic and heroic (always white)^(14)^ women striving toward an ideal of perfection, while social inequality, the racialized origins of the women who have historically provided care, and the concrete reality of nursing labor clearly demonstrate the opposite, why should we expect “others” to see us differently? As long as we camouflage the harsh realities of nursing work with projections of purity and gender stereotypes^(15)^, precarious working conditions, devaluation, invisibility, and every form of violence will continue to characterize the social reproduction labor performed by nurses and nursing technicians^(10,11,12,13)^.

Furthermore, while some nurse scholars devote themselves to moralizing discourses that oppressively burden the profession, researchers from other fields have produced precise critiques of the ideological, eugenic, elitist, and racist legacies of nursing professionalization^(7,8)^, which certainly contribute to the “overexploitation” of labor within the neoliberal context of the Unified Health System^(13)^. It is therefore time to listen carefully to “society”, represented here by the insightful critiques offered by researchers from other fields, instead of hiding behind unfounded analyses. It is our responsibility, as nurses, to build the political agency of our own history, which necessarily includes criticism and self-criticism of the science we produce. This theoretical study is intended precisely to address this gap in reflective scholarship on care and nursing.

Regarding the other challenges listed above, we discuss the incongruities concealed beneath the fragile “veil” of loving care, which we confront through Nancy Fraser’s perspective^(5)^. Beginning with the well-recognized difficulty of defining care within nursing science, a literature review of 39 studies identified several attributes commonly associated with care in the nursing literature, broadly characterized as “therapeutic communication”, “bedside presence”, “empathy”, “scientific care”, “subjectivity”, “promoting well-being”, and the phrase “science, art, and creativity”. Other attempts to map the concept have also described care as a “moral ideal”, an “ethical practice”, an “existential experience”, a “practice of loving kindness”, or a “spiritual practice”^(3)^. Unsurprisingly, much of this body of work is derived from the Theory of Human Caring, recently revisited^(1,4)^. A quantitative study identified 79 publications on this theory, involving 221 authors and 155 institutions from 18 countries across all continents, attesting to the predominance of care as “love” within the scientific discourse of nursing^(2)^.

Paradoxically, none of the reviewed publications^(2,3)^ conceptualizes care within the socioeconomic context of concrete human social relations, whether as a helping action shaped by the micropolitics of power relations^(17)^ or within the sphere of social reproduction labor, which is our focus here. This means that the material activities involved in care, unequally performed by women in nursing, remain largely absent from the concerns of mainstream nursing thought. Certainly, where there is power, there is resistance. Consequently, the theoretical current that mythologizes “love” is far from representing the only perspective in the scientific discourse of nursing; rather, it is simply the most idealized, sentimental, and politically harmful to the profession. In this regard, we identified powerful, relevant, and consistent critiques within both the international and Brazilian nursing literature that challenge these interpretations through robust engagement with Feminist Political Economy (Social Reproduction Theory [SRT]) and black feminist thought, among other perspectives, with which we engage in dialogue^(12,17,18,19,20)^.

In relation to this productive intersection between nursing and feminist epistemology, SRT, a reinterpretation of Marx’s concept of labor, has renewed theoretical interest in the field in light of the structural crisis of neoliberalism. From a feminist perspective, the critique focuses on the unsustainable division imposed by capital between productive (economic) labor, i.e., labor that generates value through commodity production, and reproductive (social) labor, which produces and sustains the social conditions required for the renewal of the labor force. In other words, the reproduction of workers encompasses not only their biological reproduction but also the fulfillment of their vital needs, as well as the institutions, symbols, and power structures that sustain society in the broad sense of social reproduction^(21)^.

A productive debate exists among feminist scholars regarding whether reproductive labor generates economic (exchange) value, given that the labor force itself, reproduced through care, is a commodity. The central question is as follows: does the product generated by care work, i.e., human labor power, constitute a commodity with value that produces surplus value (abstract labor) through exchange? Two major perspectives have emerged: i - the Italian workerist feminists argue that value is also produced outside formal workplaces, namely within the private domestic sphere (the so-called global social factories). Consequently, they contend that surplus value is socially embedded in human labor power. Thus, the relationship established between the male worker and the capitalist already incorporates the surplus value generated by women, even though it remains unpaid; ii - in contrast, the Anglo-Saxon tradition, to which Nancy Fraser belongs, argues that reproductive labor does not produce exchange value but rather something fundamental: the conditions that make all extraction of surplus value possible. According to this perspective, value is the expression of a social relation produced within labor organized under capitalist conditions (industrialized, specialized, fragmented, and alienated). Therefore, care work (such as domestic labor) produces use values that are consumed in the reproduction of labor power but does not generate surplus value in the productive sense. The difference lies in the interpretation of the category of “value”, a controversial issue within Marxism itself, the detailed discussion of which is beyond the scope of this article^(21)^.

Nevertheless, regardless of how feminist scholars interpret Marx’s theory of value, both perspectives challenge the unjust conditions under which women perform care work within the patriarchal, sexist, and racialized relations of capitalism–this being the primary focus of this study. This discussion is equally relevant to nursing science, given the important critical scholarship on Marx’s labor theory of value produced since the 1980s (and more recently expanded), a singular period in Brazilian nursing during which theoretical reflection coincided with political engagement concerning the profession’s historical development. In our view, although discussions of the labor-value category in nursing have not sufficiently addressed gender issues within the profession, dialogue with feminist epistemology can foster productive debates and critical tensions regarding nursing labor, both in the sphere of social reproduction and within the health production complexes, where nursing constitutes an overexploited labor force^(11,12,13,22)^.

Returning to the context of SRT, reflection on labor through this reinterpretation of Marx lies at the heart of the contradiction identified by Fraser^(5)^, namely that care work, which sustains the social conditions allowing people to be exploited by capital, is itself subordinated by capitalism, even though capitalism intrinsically depends on the very care work performed by women, whether nurses or not. Thus, while capitalism depends fundamentally on care, it simultaneously devalues, precarizes, “cannibalizes”, dispossesses, or fails to compensate reproductive labor, particularly when historically shaped by race and social class^(5,6,21)^.

Given the structural, material, sociohistorical, and cultural realities of capitalism, the “loving” conception of care endorsed by scholars who claim to embody the profession’s historical legacy^(1,2,3,4)^ merely reinforces the “domestication” (disciplining) of women for a double or even triple form of capitalist exploitation. Accordingly, choosing SRT, according to Nancy Fraser, as the analytical framework is justified because it enables us to unveil the many dimensions of the exploitation of nursing labor within capitalist relations, both in its most visible form through wages and in the invisible layers concealed by uncompensated dispossession or by the symbolically romanticized domestication of women’s labor. Let us explain how this occurs in nursing. The first dimension involves exploitation through the formal employment of nurses and nursing technicians in institutions where work is underpaid, precarious, overexploited, undervalued, and harmful to workers’ health^(10,12,13,14)^. The second and third forms of appropriation occur through the unpaid care work carried out by women in their homes, rendered invisible by the sentimentalized ideology that women are “born to care”^(15)^. As for these forms of expropriation within the public sphere, recent studies have shown that black women in nursing experience dispossession (the theft) of their working time, as they work 26 hours and 8 minutes more than white men and 21 hours and 20 minutes more than white women in the profession, despite receiving the same monthly pay^(12)^.

We find, therefore, that we become easy targets for the domestication of reproductive labor under capitalism whenever we reaffirm discourses that idealize care, treating it not as both abstract and concrete labor, historically embedded in the social relations of production and reproduction, and therefore capable of being transformed through the profession’s political struggle, but rather as a “moral duty”, an “ethical obligation”, an “existential experience”, an “immutable essence”, or “compassion and a loving practice”, supposedly grounded in women’s maternal instinct, a notion reproduced by part of the scientific literature and echoed in nursing practice^(1,2,3,4,7,8,9,15)^.

Given the influence of the discourse of “love” on the profession’s political and economic fragility, this study addresses the following question: what contributions does Nancy Fraser’s conception of care as social reproduction labor offer to confronting the self-discourse of “love” in nursing? We argue that care as social reproduction labor–purposeful labor aimed at meeting the social needs of the labor force in capitalist societies–reveals the weaknesses of the self-discourse of “love” in nursing by exposing the material, intersectional, capitalist, and sociohistorical conditions that sustain it, thereby supporting counter-hegemonic discursive practices within the profession. Accordingly, the objective of this study is to critically discuss the contributions of Nancy Fraser’s conception of care as social reproduction to confronting the self-discourse of “love” in nursing.

## METHOD

This is a theoretical study grounded in SRT, according to Nancy Fraser, which represents a feminist perspective within Critical Theory for unveiling the oppressive dimension of the self-discourse that frames care as “love” in nursing science. We primarily adopted Fraser’s reflections on the contradictions between care and capitalism^(5,6)^, as they contribute to a contextual understanding of care relationships and to questioning the discursive practices that shape the education and professional practice of women in nursing. The theoretical investigation of SRT is part of a broader research project that analyzes care from the perspective of feminist epistemology and supports undergraduate and graduate courses, as well as the supervision of research subprojects addressing gender issues in nursing. As a limitation, given the restricted space available and the number of theoretical categories mobilized in this study, we acknowledge the introductory nature of the present text, which should be understood as an initial problematization open to future developments (including the incorporation of empirical sources) and intended to stimulate reflective debate among peers.

To expose the discourses of “love” that so profoundly oppress us within the profession, we adopted the theoretical-methodological framework of the negative trihedron of care, grounded in the methodological framework of depth hermeneutics for ideology analysis and adapted to the discursive practices of care. The negative version of the trihedron of care is based on Theodor Adorno’s negative dialectics and is structured around three moments: neglecting care (*descuidar*), confronting (*confrontar*), and unsettling (*arrepiar*)^(17)^. Critique begins with neglecting care, i.e., with denying what initially appears to be self-evident. Thus, we deliberately set aside conceptions of care embedded in precepts, dogmas, or romanticized notions of the illusory concept of “love”–a term that encompasses all the related descriptors discussed previously. Once the nursing self-discourse has been negated, we confront these interpretations with their own foundations and with the concrete reality of nursing work (which corresponds to the immanent critique of negative dialectics), contrasting what is said with what actually occurs in the daily lives of nurses and nursing technicians. Unsettling, the third moment of this deconstructive process (adapted from Adorno’s notion of “shuddering”), does not provide definitive answers. Rather, it intensifies the discomfort, sometimes even physical discomfort, generated by the discursive analysis, expanding opportunities for critical questioning while preserving the negativity inherent to critique.

The article is organized into three sections. First, we reflect on the contradiction between care and capitalism according to Nancy Fraser, reconstructing her argument that conceives care as social reproduction labor. Second, we undertake an immanent critique of the nursing self-discourse of love through the negative trihedron of care. Finally, we recalculate alternatives for a critical pathway concerning care work in scientific thought, nursing education, and the profession’s political practice. All translations cited in this article are our own.

## THE CRISIS OF CARE ACCORDING TO NANCY FRASER: CONTRADICTIONS BETWEEN CAPITALISM AND SOCIAL REPRODUCTION LABOR

Understanding care in capitalist societies requires moving it beyond the moral, emotional, or vocational sphere and situating it as labor within the social relations that sustain both the reproduction of life and capital accumulation. This reflection extends beyond a material perspective by proposing a broader understanding of capitalism as an institutionalized social order that depends on noneconomic conditions, such as those created through social reproduction labor^(5,6)^. Another necessary distinction concerns the concepts of social reproduction and care work^(21)^. Social reproduction encompasses the daily and intergenerational processes that produce human beings and their social bonds, including culture and social institutions. Care work, in turn, constitutes one dimension of social reproduction characterized by activities directly aimed at maintaining people’s social well-being, such as caring for older adults and children, maintaining households, and sustaining communities. Thus, all care work is part of social reproduction, but social reproduction cannot be reduced to care work alone, since it also includes other dimensions, such as education, socialization, and the symbolic organization of social life. Feminist critiques of Marxism focus on gender inequalities under capitalism, given that the labor required to sustain life disproportionately falls upon women. The paradox under scrutiny is this: although care activities are indispensable to society, capitalism treats them as free, natural, and external to the economy, thereby concealing the exploitation of labor^(5,6,21)^.

Within capitalism, a reductionist semantic inversion occurs between labor and employment, with the latter representing only a small portion of the former, characterized by the formal and paid relationship of capitalist production. As a result, social reproduction activities aimed at meeting human needs are regarded as unproductive because they supposedly do not generate economic value in the capitalist sense (commodities), even though they constitute the preconditions for all capitalist exploitation. According to Nancy Fraser, this separation between the social (reproductive) and economic (productive) spheres was artificially constructed by capitalism, giving rise to a structural contradiction whereby capitalism depends on social reproduction labor while simultaneously rendering it invisible, devaluing it, and “cannibalizing” it. This contradiction results in the care crisis, which devours ecology, political power, and social bonds, while colonizing ethnic groups, racializing, and gentrifying social reproduction^(5)^. Since the consolidation of industrial capitalism in nineteenth-century England, coinciding with Victorian morality, the division associating productive labor with commodity production and reproductive labor with the domestic sphere has ignored the fact that social reproduction labor produces what makes capitalist exploitation possible: human beings themselves. The separation between production and reproduction also has a gentrified character, insofar as care work has been regarded as women’s “duty” within patriarchal societies. This process not only naturalized women’s responsibility for care but also institutionalized new forms of subordination: the exploitation, dispossession, and domestication of labor, all intertwined within the ideology of “love”^(5,6)^.

In this context, exploitation refers to the extraction of surplus value from wage labor. Under this system, the doubly free worker receives wages equivalent to the cost of reproducing their labor power (that is, only enough to remain alive and continue being exploited), while capital appropriates the surplus value produced. Workers are considered “doubly free” because they are free to sell their labor power but also because they do not own the means of production, thereby becoming entangled in the contradictory relations of exchange. In other words, this process takes place under the appearance of a free exchange of equivalents while actually involving the systematic appropriation of surplus labor time by capital. Dispossession, in turn, consists of the direct and coercive seizure of resources, land, and labor power that enables the expansion of capitalism, including the colonization of peripheral countries such as Brazil. Unlike exploitation, which presupposes formally free workers, dispossession operates through violations, outright confiscation, and plunder, frequently associated with processes of racialization, colonialism, and political subordination. Moreover, dispossession is neither an obsolete phenomenon nor one confined to the origins of capitalism; rather, it is a structural characteristic that reduces production costs in order to sustain capital accumulation at the expense of subjugated, racialized, and migrant populations. Unlike formally exploited labor, dispossession does not cover the costs of sustaining the lives of racialized men and women who constitute the cheap, subordinate, and precarious labor force^(6)^.

The third dimension, the domestication of labor, refers to the set of life-sustaining care activities that have historically been sentimentalized as love and assigned to women. Consequently, these activities have become invisible, devalued, and frequently unpaid labor. The term “domesticated” does not refer to the domestic sphere itself but rather to a historical process of disciplining women’s bodies and minds. It also encompasses the naturalization of care activities, which come to be perceived as expressions of affection, moral obligation, or vocation rather than as labor. Clearly, these three dimensions, exploitation, dispossession, and domestication, do not operate independently but rather in an integrated manner, constituting a system for appropriating labor and life. While exploitation extracts value from wage labor and dispossession provides resources and reduces production costs, domestication disciplines bodies, paving the way for both symbolic and material appropriation. From this perspective, capitalism is sustained not only through formal economic exploitation but also through the systematic and domesticated appropriation of social reproduction labor, which is treated as an inexhaustible natural resource despite being embedded in the contradiction between capitalism and care^(5,6)^.

Understanding care as social reproduction labor makes it possible to situate it at the center of capitalism’s crisis and to demonstrate that it is not merely a complement to the extraction of surplus value but rather its very condition of possibility. Therefore, care does not constitute an ethical, emotional, “benevolent”, or relational dimension of social life (or of women’s lives). Instead, it is a structural element of political economy whose erasure serves the interests of capitalist accumulation. Viewing care as social reproduction labor also makes it possible to expose the devaluation of women’s labor in nursing, since historical conditions depend on how we position ourselves in relation to, confront, or reproduce the capitalist social order that “cannibalizes” care. In this regard, we have seen that nursing scholarship, in general, glorifies one of the principal forms of care domestication under capitalism by disguising it as “love”, embellished with moral appeals and reinforced by the authoritative discourse of science^(15)^. In contrast, in the following section, we neglect care, confront, and unsettle the conception of “love” that oppresses women in nursing.

## NEGLECTING CARE, CONFRONTING, AND UNSETTLING THE CONCEPTION OF “LOVE” IN THE PROFESSION

Let us begin by neglecting what “love” actually means within the profession’s self-discourse and why it is conflated with care. What characteristics are attributed to love? What does it reveal, and what does it conceal? Where does the premise originate that love, as well as care and the nurses who provide it, is invariably “benevolent”? What does it mean to be “good”, and who imposes this standard of goodness upon us as women? Why do we wish to appear good, charitable, altruistic, heroic, and saintly if we are human beings, made into “women”^(23)^, inhabiting bodies that desire and suffer, mortal and imperfect? Why does the explicitly romanticized discourse of “care as love” appeal to some women in nursing? What are we hiding from, and why is this discourse so seductively comforting to our ears? Many of these questions lie beyond the scope of this article, yet we raise them as an exercise in reflective neglecting care. Let us focus, then, on the main point: what is said about “love” and its meanings within nursing.

As with the conceptual ambiguities surrounding care, love itself is not treated as an object of theoretical reflection or conceptual development within nursing science. Instead, it is invoked as a self-evident form of “kindness”, according to which “love and care are two universal givens”^(24)^, a dogmatic assertion presented as immune to criticism. The terms “care”, “love”, and “nursing” are treated as interchangeable, regarded as synonymous with purely moral attributes, assumed to constitute an “ideology of caring” and “an ideal and value of care [...] that must become a desire”. Some of these conceptual imprecisions are illustrated by the expression “Caritas” (derived from the lexical root meaning “charity”), presented as an “explicit connection between care and love”^(4)^ and also described as “timeless”, the “core of science and nursing”, the “most astonishing and mysterious cosmic forces”, and the “essence of nursing and the most central and unifying focus of practice”. Care, nursing, and love become so thoroughly intertwined that, according to these authors, “we must impose upon our own behavior, rather than upon that of others, our will to care and to love”^(24:62)^. In the same assertive tone, the “Clinical Caritas Processes” summarize the commandments of nursing care, including “forgiveness”, the altruistic values of kindness, empathy, concern, and love, acceptance, and “openness to miracles”^(4,24)^, a vocabulary strikingly similar to the Catholic Decalogue.

The discourse of love, care, and nursing generates illusions of perfectibility, divinization, asceticism, and the “disembodiment as a supposed superiority of nurses”, as stated in one of the most incisive critiques produced by a scholar in the field^(25)^. Indeed, the hyperbolic self-exaltation of nurses runs the risk of descending into delusion. Nursing practice and its practitioners are described as “a mystery to be lived”, existing “beyond what can be objectified” and “concerned with hidden truths”. Nursing is no longer understood as work historically situated in time and space (and therefore subject to pressures for change), but rather as “a philosophical concept that suggests tenderness” and “passion”, “offering the promise of preserving the human being in society”. Nurses–never gendered in the language used–are portrayed as those who “see the world differently from the medical-natural science paradigm” because they care for “the wholeness of the human personality”. They are spiritually evolved beings who “creatively transcend themselves” and who should “consider certain metaphysical and moral ideas as guides for their own endeavors”. It is further claimed that nurses are the “only ones who hear and honor another person’s story and the full intensity of their feelings”, because they “can gain indirect access to a person’s mind and inner self”, thereby being “capable of forming a union with another person at a level that transcends the physical”^(4,24:89)^.

Readers will likely agree that this view of nursing is completely disconnected from the reality of care work and, moreover, is politically harmful to efforts aimed at confronting social inequalities within the profession^(10,11,12,13,14,15,16,17,18,19,20)^. Claiming that it is merely an “ideal”, acknowledged to be abstract, does not exempt advocates of “love-care” from responsibility for its oppressive consequences in the concrete reality experienced by nurses and nursing technicians, the majority of whom remain shaped by the myth of maternal/romantic love^(5,6,21,23)^. Clearly, both the theory and the charitable processes of care are inapplicable in professional practice^(2,3,25)^, given the evident degree of fantasy underlying these conceptions. Critical perspectives on this theoretical approach argue that care-as-love is “only one ideal among many”, politically unrealistic, and that it ignores while simultaneously reinforcing the powerful structures of power that operate against us. These critiques further argue that this paradigm of care was conceived to be “ineffable”, permanently allusive, eager to exercise power over others, and intended to inflate the profession’s self-esteem^(25)^.

In agreement with these counterarguments, we highlight the understanding of care as social reproduction labor, adopted here as an expression of the critical-discursive act of neglecting care in relation to the fallacies of mythologized love within capitalist relations. We adopt the feminist interpretation of love as a mythologized ideology that symbolically victimizes women and reinforces male domination^(5,6,21)^. Under patriarchy, love as libidinal investment is structured to discipline women’s bodies, shaping them to remain subordinated as the “second sex” (object), while the “first” belongs to the masculine universe (subject)^(23)^. From this perspective, given the disparity between the pretentious ideal of “love-care” and the degrading working conditions experienced by nurses and nursing technicians providing direct care, one must ask how ideals of purity and perfection became so deeply internalized within the profession. Nancy Fraser’s sociohistorical contextualization of “cannibal capitalism” offers an important clue. Three historical developments that marked the consolidation of capitalism in the nineteenth century became disastrously intertwined with the professionalization of modern nursing: i - the strengthening of industrial capitalism and England as the center of political and economic power; ii - Victorian morality; and iii - the English feminist movement against the Contagious Diseases Acts. Let us examine more closely how these historical threads converge in professional nursing.

To begin with, the strengthening of industrial capitalism reinforced the separation between productive labor (economic, masculine) and reproductive labor (social, feminine), made possible through the colonization and domestication of women as “housewives” and “angels of the house”. Within this context, efforts to guarantee social reproduction through unpaid labor became deeply intertwined with male domination. In England, and this is the second point, the rise of capitalism and male domination coincided with the puritanism of the Victorian era and the emergence of the Nightingale model of nursing, whose influence spread worldwide. In this context of moral austerity, the consolidation of industrial capitalism affected poor and wealthy women differently, generating dissatisfaction on both sides. Poor racialized women did not conform to Victorian ideals of domesticity (modesty, submission to husbands or fathers, confinement to domestic tasks or activities considered feminine, and regulation of the body and social behavior). Wealthy women, on the other hand, such as Florence Nightingale, enjoyed material comfort and moral prestige but demanded freedom and legal autonomy. According to Nancy Fraser, the “solution” to the separation between productive and reproductive labor came at women’s expense by placing them in opposition to one another. The conflict lay in the fact that the philanthropic concerns of upper- and middle-class Victorian women diverged from the material interests of their “fallen sisters”, a divide further intensified by moral judgments concerning prostitution^(5)^.

The moral confrontation among women during the Victorian era reached its peak in the struggle for the repeal of the Contagious Diseases Acts, led by the feminist movement known as the “Women’s Revolt” (1864–1869), the third historical element shaping the development of nursing^(26)^. The purpose of these Acts was to control sexually transmitted infections among military personnel by subjecting women suspected of prostitution to forced gynecological examinations and compulsory detention. Curiously, although for opposing reasons, this offensive legislation united women from different social classes, led by the feminist movement of the Ladies National Association, which denounced the Acts as discriminatory and violent. Sex workers, who were the primary victims, also rebelled against the abuses they suffered. Some aristocratic women joined the movement for philanthropic reasons, whereas others, such as Florence Nightingale, did so for moral reasons, arguing that the legislation “officially sanctioned the vice” of prostitution. Although the Acts were eventually repealed, the consequences of this episode reinforced norms of social purity and granted new police powers to monitor the working class^(26)^.

However, how could the professionalization of modern nursing have been influenced by historical contexts that appear to be so distinct? In our view, the rise of capitalism, the power of the aristocratic class, and the values of whiteness and social purity that it promoted were incorporated into the Nightingale model, in which lady nurses (registered nurses) were supposedly endowed with a status of moral superiority over nurses (nursing technicians), resulting in the subjugation of women and the surveillance of their bodies. The literature has extensively discussed how this moral model of the “ideal nurse” shaped nursing education worldwide, including in colonized, racialized, and brutally dispossessed countries such as Brazil^(7,8,9)^. The moralizing and scientistic solution offered by the Nightingale model failed to resolve the contradictions experienced by real women with bodies, race, and social class. Consequently, inequalities and oppression persisted both within and beyond the nursing workforce^(10,11,12,13)^. Nevertheless, the disciplining of women, who constitute 80% of the health workforce, required one additional ingredient, the most powerful of all, embodied in the image of the “white angel”, the mythical face of the “nursing-care-love” triad. Indeed, the misleading image of nursing as self-sacrificing purity is nothing more than the transplantation of the “angel in the house” ideology into the hospital setting, thereby naturalizing the separation between capitalist production and reproduction^(5,7,8)^.

In light of this historical trajectory, we observe that the notion of “care-as-love” continues to prevail in scientific discourse, nursing education, and professional practice, resulting in political fragility and persistent inequities. One illustration of the profession’s moral authoritarianism is the assertion that “we must impose upon our own behavior [...] our will to care and to love”^(24:62)^, a faithful representation of the domestication of nursing labor^(5,6)^. Nursing care is often praised as an “obligation” or a “duty”, when it actually constitutes the dispossession of emotional labor, understood as the effort required to manage, suppress, or express specific emotions demanded in the performance of a professional role in order to meet normative expectations. Emotional labor represents one of the forms of oppression associated with social reproduction labor^(27)^. Beyond the emotional exploitation that occurs within formal employment, the prescription of care-as-love also reflects the degree of moral rigidity inherent in nursing itself. In this regard, studies have reported a high prevalence of burnout syndrome among nurses and nursing technicians, as well as exhaustion resulting from the emotional labor required by the profession. Nevertheless, neither emotional labor nor social reproduction labor is generally problematized within nursing scholarship. Instead, both continue to be regarded as intrinsic attributes of the profession, thereby renewing the oppressive power of the discourse of “love”^(11,28)^.

Once the fallacies of “love” in nursing have been discursively disregarded, despite their contrast with the profession’s harsh working reality, let us confront this self-discourse with its theoretical foundations. Let us first problematize the nonspecific and controversial “metaphysical” conception of care, at most described as a spiritual dimension that exists “outside time”^(1,4,24)^. This constitutes a gross oversimplification, supposedly grounded in Heidegger’s notion of Dasein (that which possesses the capacity to question the meaning of Being). Owing to limitations of space and purpose, it is not possible to reconstruct the entire discussion underlying such profound philosophical concepts; doing so would place us at the same level of superficiality that we seek to criticize. Nevertheless, we ask permission to delimit the core of the controversy. Metaphysics is a philosophical tradition concerned with the search for universal truths, having undergone several shifts and critiques, the most significant of which was advanced by Heidegger. His critique concerns the traditional metaphysical neglect of the question of Being (that which subsists through its meaning and cannot be grasped conceptually), in favor of entities (what we speak about, understand, and interpret; what is, as we ourselves are). From the Heideggerian perspective, by inquiring into the Being of entities through the notion of Dasein (the human being), which is open to this revelation, philosophy would overcome classical metaphysics. This would constitute a critical intervention, a “destruction” of the tradition, particularly through its innovative understanding of the temporal dimension of Being. Thus, the assertion that care is “atemporal” directly contradicts the foundational basis of Dasein, since Being exists only in time (hence the title “Being and Time”), while simultaneously placing nursing outside history and rendering it incapable of changing its own destiny^(29)^.

The second point of contention concerns the alleged representation of “Nightingale’s legacy and foundations”^(4)^ in the sentimental notion of care as “kindness”, “love”, “tenderness”, “compassion”, and related concepts. Despite the moral puritanism and Protestantism that profoundly influenced the conservatism of the profession, Florence Nightingale advocated a rational and empirical science of nursing care and rejected facile sentimentalism. Accordingly, some of her statements directly challenge the sentimental notion attributed to care-as-love. For example: “It is not the task of nursing to be kind, in the sense of doing one’s own will or gratifying one’s own feelings; it is its task to carry out the laws of health”; or again: “a nurse is not a devoted and obedient woman, but should be a thoughtful and educated person”^(30:200)^. Thus, the purported representation of Florence Nightingale’s ideas in the notion of care as love collapses because it contradicts the very foundations of her thought.

By neglecting and confronting the self-discourse of love, we arrive at a moment of unease, when we allow ourselves abstraction, discomfort, questioning, criticism, and self-criticism. Let us return to our initial questions, especially why the romanticized discourse of “love” appeals to some women in nursing or why it sounds so seductively comforting to our ears. Perhaps the hidden pain that we soothe through self-deception is the same pain that unites us in the condition of “woman” as the “Other” of the universal “Self”, relegated to the status of an object named by the authority of the male subject. In the second volume of *The Second Sex*, Simone de Beauvoir discusses women’s experiences of love, what they make of their condition, how they experience it, and the forms of escape available to them. Through the interconnections among the categories of the “narcissistic woman” (for whom self-love is an absolute end), the “woman in love” (the mythification of romantic love), and the “mystic” (the surrender and devotion of love to God), the philosopher reflects on the circumstances that lead women to turn inward and dedicate their love as a form of compensation for what has been denied to them as subjects^(23)^.

In keeping with Beauvoir’s reflections, it appears that the need for self-affirmation, moral superiority, and immutable identity expressed in nursing discourse intersects with the latent pain of the “Other” shared by all of us, women conditioned by time and by what we do within it. Furthermore, “these efforts at salvation can only end in failure; either woman enters into a relationship with something unreal, her double or God, or she creates an unreal relationship with a real being”^(23:876)^.

## FINAL CONSIDERATIONS

We need to discuss care as social reproduction labor in nursing, while also incorporating a gender perspective and fostering meaningful dialogue with feminist epistemology in undergraduate and graduate curricula, with repercussions for scientific production and the political practice of the profession. Rather than pursuing the unlikely search for universal truths or esoteric mysteries that contribute neither to praxis nor to the critical profile outlined in academic curricula, let us broaden the visibility of the critical and self-critical resistance embodied in the scientific production of nurse researchers. In doing so, we unveil the totalitarian discourses of “love” that imprison our reflective action and our capacity to shape the history of the profession. The science we produce must problematize the social and gender contradictions within nursing, whose labor has been rigidly domesticated in the education of nurses and nursing technicians. Let us likewise challenge the alleged neutrality of science, intertwined with the self-centered pursuit of evidence as a discourse of authority, exposing its ideological commitment to the sexist, misogynistic, patriarchal, racist, and anti-ecological capitalist social order that cannibalizes us beneath false songs of nobility. The knowledge we produce cannot exempt itself from its political dimension or from its commitment to improving working conditions, promoting fair remuneration, and confronting violence within the profession, among other urgent priorities.

For a critical path toward nursing care, let us finally recognize that our greatest strength lies precisely in what has been systematically erased by the conservatism and elitism of modern nursing: the diversity of cultures, genders, sexualities, races, social classes, and forms of knowledge embodied by the women who work tirelessly for the health of the Brazilian population across the different spaces of social reproduction, not only in direct care. Perhaps then we may inaugurate other forms of love, more closely aligned with the political and economic dimensions consistent with women’s democracy.

## Data Availability

The entire dataset supporting the results of this study was published in the article itself.
