# Supplementary material for: It is not love, but social reproduction labor: nursing care in light of Nancy Fraser
Source: Rev Esc Enferm USP. 2026 Sep 28;60:e20260127. doi: 10.1590/1980-220X-REEUSP-2026-0127en (PMC13622891; doi:10.1590/1980-220X-REEUSP-2026-0127en)
Supplement: Translation

# Translation: Não é amor, mas trabalho de reprodução social: cuidado e enfermagem à luz de Nancy Fraser

**DOI:** 10.1590/1980-220X-REEUSP-2026-0127pt

**Authors:** Maria Raquel Gomes Maia Pires, Gabriela Duarte Almeida Mundim

**Affiliations:** 1 Universidade de Brasília, Faculdade de Ciências da Saúde, Departamento de Enfermagem, Brasília, DF, Brasil.; 2 Universidade de Brasília, Centro de Estudos Avançados Multidisciplinares, Brasília, DF, Brasil.

**Keywords:** Estereótipo de Gênero, Enfermagem, Cuidado de Enfermagem, Discurso, Feminismo, Trabalho

## Abstract

Estudo teórico que adota a concepção de cuidado como trabalho de reprodução social em Nancy Fraser como crítica do autodiscurso científico do cuidado como “amor” na enfermagem. Argumentamos que o cuidado como trabalho de reprodução social, ação esforçada que visa atender às necessidades sociais da força de trabalho nas sociedades capitalistas, expõe as fragilidades do “amor” da enfermagem – por desnudar as condições materiais, interseccionais, capitalistas e sócio-históricas que o sustentam, subsidiando práticas contra-hegemônicas. Inicialmente refletimos sobre a contradição entre cuidado e capitalismo. Em seguida, procedemos à crítica imanente do autodiscurso do amor na enfermagem por meio do triedro negativo do cuidar. Na conclusão, recalculamos alternativas para uma rota crítica do trabalho do cuidado na profissão. Propomos a inclusão da perspectiva de gênero e o diálogo com a epistemologia feminista nos currículos da graduação e da pós-graduação, com repercussões nas produções científicas e na prática política da enfermagem. A ciência que produzimos há que problematizar as contradições sociais e de gênero na enfermagem, cujo trabalho tem sido rigidamente domesticado.

## INTRODUÇÃO

A concepção de cuidado no discurso científico da enfermagem, seja na formação, na prática ou nas organizações de classe em geral, historicamente se confunde com dever ou atributo moral, fundado em equívocas noções de bondade, amor, altruísmo e benevolências afins que supostamente elevariam a nobreza da profissão. Há um autodiscurso de parte das enfermeiras pesquisadoras que posicionam o cuidado como “prática amorosa”, “compaixão”, “a essência e o núcleo disciplinar” da profissão, por vezes como se fossem termos intercambiáveis, isto é, como se cuidado e enfermagem fossem sinônimos^(1,2,3,4)^. De modo mais evidente, teóricas da enfermagem evocam “explicitamente o amor e o cuidado, buscando a fusão para um paradigma expandido que se conecte com as dimensões espirituais e os processos vitais das experiências humanas”^(4:132)^.

Em contraponto a essa vertente teórica que se diz representante “[…] da herança e dos fundamentos de Nightingale”^(4)^, concebemos o cuidado na materialidade das relações sociais de produção, ou seja, como trabalho de reprodução social na acepção de Nancy Fraser, entendido como conjunto de ações esforçadas que visam atender às necessidades sociais da força de trabalho (de alimentação, abrigo, moradia limpa, descanso, bem-estar, saúde, educação, outras) nas sociedades capitalistas. O trabalho reprodutivo é marcado por condições interseccionais que exploram, espoliam e domesticam (disciplinam) injustamente o trabalho das mulheres, incluindo a enfermagem, mas também de outras que assumem as tarefas domésticas, cuidam de filhos, idosos e afins^(5,6)^. O entendimento do cuidado como trabalho de reprodução social do capitalismo não se restringe a dimensão assistencial da enfermagem, mas envolve todas as ações pertinentes a atuação das enfermeiras e técnicas no âmbito das políticas públicas de saúde e de educação (gestão, educação, pesquisa, assistência), em sentido amplo, como processo de reprodução material e simbólica da força de trabalho no capitalismo. Neste estudo, utilizamos essa concepção de cuidado como crítica do discurso ilusório da profissão, quer dizer, daquele que concebe o cuidado como “amor” e adjetivos correlatos, brevemente descritos acima e problematizados nos parágrafos seguintes.

De início, em sintonia com as reflexões da epistemologia feminista sobre as violências interseccionais de gênero, raça, classe, sexualidade e etarismo sobre nós, mulheres, enfermeiras ou não, questionamos se todas as pessoas que realizam o trabalho do cuidado são (ou precisam ser) enfermeiras, como parecem sugerir as teóricas da enfermagem^(1,2,3,4)^. Pensemos juntas: o que ganha a profissão em tomar heroicamente para si, como uma controversa “essência” imutável^(1,4)^, todas as violências simbólicas, institucionais e estruturais envolvidas na responsabilização desigual das mulheres pelo trabalho do cuidado? Não seria politicamente mais estratégico se fizéssemos o justo oposto, ou seja, enfrentássemos endogenamente a falácia opressiva dos enunciados que apenas docilizam corpos e nebulizam o pensamento crítico das mulheres na enfermagem? Decerto, essa é apenas uma das questões, mas há muito mais em jogo quando reeditamos hegemonicamente os discursos ideológicos do “amor” na enfermagem. Expliquemos melhor.

O discurso da suposta “bondade amorosa” do cuidado inviabiliza o desvelamento e o enfrentamento coletivo das contradições encrustadas na profissão, dentre elas: i – as fragilidades epistêmicas sobre o cuidado e os esforços insustentáveis para torná-lo um “ideal moral” ou uma “ciência unitária” de “amor infinito”, imune à crítica^(1,2,3,4)^; ii – a nefasta influência da branquitude, da origem eugênica, racista e classista da profissionalização da enfermagem, produtora de práticas discursivas que ratificam endogenamente opressões contra nós, mulheres^(7,8,9)^; iii – a superexploração do trabalho da enfermagem no contexto capitalista, os baixos salários e os muitos adoecimentos de enfermeiras e equipe, por vezes naturalizados como dever do ofício, altruísmo ou pretensos “heroísmos”^(10,11,12,13,14)^; iv – a violência simbólica dos discursos científicos estereotipados e moralizantes, perpetrados acriticamente na formação e na prática política da categoria^(15)^; v – a equívoca visão de que seria “a sociedade” que estigmatizaria e desvalorizaria a profissão, como se a enfermagem não fizesse parte e não contribuísse para a forma como somos retratadas^(16)^.

Um olhar atento sobre a influência do “processo cáritas”^(1,4)^ na enfermagem evidencia facilmente que a sociedade nos enxerga exatamente como insistimos hegemonicamente em nos apresentar (ou negar), parte dela que somos. Referimo-nos aqui aos confortáveis argumentos de que é a sociedade que desvaloriza e defere estereótipos de gênero contra a profissão, como se estivéssemos fora dela^(14,16)^. Entretanto, se o discurso prevalente e contraditório de boa parte das cientistas enfermeiras insiste em afirmar que somos seres moralmente superiores, proprietárias do cuidado como uma essência “unitária” da profissão, altruístas e heroínas (sempre brancas)^(14)^ em busca de um ideal de perfectibilidade, quando a desigualdade social, a origem racializada das mulheres que sempre cuidaram e o trabalho concreto da profissão evidenciam o claro oposto, por que esperar que “os outros” nos vejam de forma diferente? Enquanto camuflamos a dura realidade de trabalho da profissão com projeções de desejos de pureza e estereótipos de gênero^(15)^, sobram precariedades, desvalorização, invisibilidades e violências de toda sorte na realidade do trabalho de reprodução social das enfermeiras e técnicas de enfermagem^(10,11,12,13)^.

Ademais, enquanto parte das estudiosas enfermeiras se ocupa com moralizações que recaem opressivamente sobre a profissão, pesquisadoras(es) de outras áreas realizam críticas precisas sobre as heranças ideológicas, eugênicas, elitistas e racistas da profissionalização da enfermagem^(7,8)^, o que certamente contribui para o caráter de “superexploração” do trabalho no contexto neoliberal do Sistema Único de Saúde^(13)^. Passa da hora, então, de escutarmos atentamente “a sociedade”, ora representada na crítica arguta dos outros pesquisadores sobre nós, ao invés de nos escondermos em análises infundadas. Cabe a nós, enfermeiras, construirmos a agência de luta política da nossa história, que inclui a crítica e autocrítica da ciência que produzimos. Este estudo teórico se insere justamente nessa lacuna de produção reflexiva sobre o cuidado e a enfermagem.

Sobre as demais dificuldades da profissão acima elencadas, comentemos as incongruências encobertas pelo frágil “véu” do cuidado amoroso, contra as quais nos contrapomos com Nancy Fraser^(5)^. A começar pela reconhecida dificuldade em conceituar o cuidado pela ciência da profissão, uma revisão da literatura de 39 estudos identificou alguns atributos do cuidado na produção da enfermagem, caracterizados genericamente como: “comunicação terapêutica”; “presença ao lado do leito”; “empatia”; “cuidados científicos”; “subjetividade”; “ajuda ao bem-estar”; até o jargão “ciência, arte e criatividade”. Nas demais tentativas de mapeamento, figuram ainda “ideal moral”, “prática ética”, “experiência existencial”, “prática da bondade amorosa” ou “prática espiritual”^(3)^. Evidentemente, grande parte dessas produções são devedoras da Teoria do Cuidado Humano da enfermagem, revisitada recentemente^(1,4)^. Um estudo quantitativo identificou 79 produções sobre essa teoria, correspondente a 221 autoras(es) e 155 instituições de 18 países, distribuídos em todos os continentes, o que atesta a força preponderante do cuidado como “amor” no discurso científico da profissão^(2)^.

Paradoxalmente, em nenhuma das produções revisadas^(2,3)^ o cuidado é conceituado no contexto socioeconômico das relações sociais concretas humanas, seja como ação da ajuda tensionada pela micropolítica das relações de poder^(17)^, seja na esfera do trabalho de reprodução social, que ora nos interessa, o que significa que as atividades materiais do cuidado, desigualmente realizadas pelas mulheres na enfermagem, passa ao largo nas preocupações do pensamento hegemônico da enfermagem. Certamente, se há poder, há resistência, por conseguinte, a corrente teórica que mitifica o “amor” está longe de significar o pensamento único no discurso científico de enfermeiras; ele é apenas o mais idealizado, sentimental e danoso para a ação política da categoria. A esse respeito, identificamos críticas potentes, relevantes e consistentes na produção científica da enfermagem que confrontam essas versões, em todo o mundo e nacionalmente, com rica ancoragem na Economia Política Feminista (Teoria da Reprodução Social [TRS]) e no pensamento feminista negro, para citar algumas, com as quais dialogamos^(12,17,18,19,20)^.

Sobre esse fecundo entrelaçar da enfermagem com a epistemologia feminista, a Teoria da Reprodução Social, uma releitura da concepção de trabalho em Marx, tem renovado o interesse de teóricas no campo, diante da crise estrutural do neoliberalismo. Sob a interpretação feminista, a ênfase da crítica recai sobre a insustentável divisão imposta pelo capital entre o trabalho produtivo (econômico), isto é, aquele que gera valor pela produção de mercadorias, e o trabalho reprodutivo (social), o qual produz e sustenta as condições sociais para reposição da força de trabalho. Noutras palavras, a reprodução da(o) trabalhadora(r) inclui tanto a dimensão biológica quanto o atendimento das necessidades vitais, bem como as instituições, os símbolos e as estruturas de poder da sociedade, no sentido amplo da reprodução social^(21)^.

Há um profícuo debate entre as feministas sobre se o trabalho reprodutivo gera ou não valor econômico (de troca), haja vista o caráter de mercadoria da(o) própria(o) trabalhadora(r) reproduzida(o) pelo cuidado. O questionamento é o seguinte: o produto gerado pelo trabalho do cuidado, a saber, a força de trabalho humana, é uma mercadoria com valor que gera mais-valia (trabalho abstrato) no processo de troca? As respostas se dividem em duas correntes, a saber: i-as operaístas italianas afirmam que o valor também é produzido fora dos locais de trabalho, isto é, no âmbito privado doméstico (as chamadas fábricas sociais globais), portanto ele contém uma mais-valia que é socialmente incorporada à força de trabalho humana. Com isso, o trabalhador homem e o capitalista contratualizam uma relação que já inclui o mais-valor gerado pelas mulheres, embora não pago; ii-por sua vez, a tradição anglo-saxônica, da qual Nancy Fraser faz parte, contra-argumenta ao afirmar que o trabalho reprodutivo não produz valor de troca, mas algo fundamental, as condições que possibilitam toda e qualquer extração da mais-valia. Nessa segunda corrente, valor é a expressão de uma relação social produzida no contexto do trabalho organizado em termos capitalistas (industrializado, especializado, fragmentado, alienado), portanto o trabalho do cuidado (como o doméstico), produz valores de uso que são consumidos para a reprodução da força de trabalho, mas não geram mais-valia, em sentido produtivo. A diferença reside na interpretação da categoria valor, algo controverso dentro do próprio marxismo, cujo aprofundamento foge aos objetivos desse artigo^(21)^.

Entrementes, seja qual for a interpretação feminista da teoria do valor em Marx, ambas contestam a condição injusta do trabalho do cuidado realizado pelas mulheres nas relações patriarcais, sexistas e racializadas do capitalismo, esse sim, o objeto prioritário de reflexão do estudo. Para a ciência da enfermagem, a discussão igualmente importa, haja vista as importantes produções críticas sobre a teoria do valor-trabalho em Marx, oriunda da década de oitenta do século XX (recentemente desdobrada), momento singular da enfermagem brasileira em que coincidem a reflexão e a prática política sobre a historicidade da profissão. A nosso ver, embora as reflexões sobre a categoria do valor-trabalho na enfermagem não discutam suficientemente as questões de gênero na profissão, o diálogo com a epistemologia feminista pode fomentar fecundos debates e tensionamentos sobre o trabalho da enfermagem, seja na dimensão da reprodução social, seja nos complexos produtivos da saúde, como força de trabalho superexplorada^(11,12,13,22)^.

De volta ao contexto da TRS, a reflexão sobre o trabalho a partir da releitura de Marx perfaz o cerne da contradição identificada por Fraser^(5)^, qual seja: o cuidado, cujo trabalho mantém as condições sociais para que as pessoas possam ser exploradas às expensas do capital, é subjugado pelo capitalismo, que depende intrinsecamente desse mesmo trabalho do cuidado realizado pelas mulheres, enfermeiras ou não. Assim, ao mesmo tempo que o capitalismo depende visceralmente do cuidado, contraditoriamente ele desvaloriza, precariza, “canibaliza”, espolia ou não paga pelo trabalho reprodutivo, sobretudo quando historicamente permeado pela raça e classe social^(5,6,21)^.

Diante da realidade estrutural, material, sócio-histórica e cultural do capitalismo, a concepção “amorosa” do cuidado, endossada por cientistas que afirmam portar a herança histórica da profissão^(1,2,3,4)^, nada mais faz do que reforçar a “domesticação” (disciplinamento) das mulheres para uma dupla ou tripla extorsão capitalista. Nesse sentido, a escolha da TRS em Nancy Fraser como referencial analítico se justifica porque com ele podemos desvendar as muitas faces da exploração do trabalho da enfermagem no âmbito das relações capitalistas, tanto na face mais visível, via salário, quanto nas camadas invisibilizadas pela espoliação não remunerada ou pela domesticação simbolicamente romantizada do trabalho das mulheres. Expliquemos como isso acontece na enfermagem. Na primeira dimensão, a exploração acontece por meio do emprego formal de enfermeiras e técnicas de enfermagem nas instituições que é mal remunerado, precarizado, superexplorado, subvalorizado e adoecido^(10,12,13,14)^. A segunda e terceira usurpação ocorrem sob a forma do trabalho do cuidado não pago realizado por nós, mulheres, nos domicílios, invisibilizado na ideologia sentimentalizada de que as mulheres “nasceram para cuidar”^(15)^. Acerca dessas formas de extorsões presentes na esfera pública, estudo recente identifica a espoliação (roubo) do tempo de trabalho de mulheres negras na enfermagem, uma vez que elas trabalham 26h08min a mais em relação aos homens brancos e 21h20min em relação às mulheres brancas da profissão, embora recebam os mesmos valores mensais^(12)^.

Constatamos, então, que nos tornamos presa fácil da domesticação do trabalho reprodutivo pelo capitalismo, toda vez que ratificamos os discursos que idealizam o cuidado, tratando-o não como um trabalho abstrato e concreto, inserido historicamente nas relações sociais de produção e reprodução, portanto modificável pela luta política da categoria, mas como um “dever moral”, uma obrigação ética, “uma experiência existencial”, “uma essência” imutável, “uma compaixão e prática amorosa”, ancorado num suposto instinto maternal das mulheres, reeditado por parte da ciência que repercute na prática da enfermagem^(1,2,3,4,7,8,9,15)^.

Diante da influência dos ditos de “amor” para a fragilidade política e econômica da profissão, o estudo tem a seguinte questão: quais as contribuições da concepção de cuidado como trabalho de reprodução social, em Nancy Fraser, no enfrentamento do autodiscurso do “amor” na enfermagem? Argumentamos que o cuidado como trabalho de reprodução social, entendido como ação esforçada que visa atender às necessidades sociais da força de trabalho nas sociedades capitalistas, expõe as fragilidades do autodiscurso do “amor” da enfermagem – por desnudar as condições materiais, interseccionais, capitalistas e sócio-históricas que o sustentam, subsidiando práticas discursivas contra-hegemônicas na profissão. O objetivo é discutir criticamente as contribuições da concepção de cuidado como reprodução social, em Nancy Fraser, no enfrentamento do autodiscurso do “amor” na enfermagem.

## MÉTODO

Trata-se de um estudo teórico fundamentado na TRS, segundo Nancy Fraser, representante da Teoria Crítica em perspectiva feminista, para desvelar a face opressiva do autodiscurso do cuidado como “amor” na ciência da enfermagem. Adotamos principalmente as reflexões sobre as contradições entre cuidado e capitalismo realizadas pela pensadora^(5,6)^, por subsidiarem a compreensão contextual das relações do cuidado, bem como para os questionamentos das práticas discursivas conformam a formação e a prática das mulheres na enfermagem. A pesquisa teórica sobre a TRS integra um projeto de pesquisa que analisa o cuidado a partir da epistemologia feminista, o qual subsidia a oferta de disciplinas na graduação e pós-graduação, bem como a orientação de subprojetos sobre as questões de gênero na enfermagem. Como limitação, em vista do espaço reduzido e da quantidade de categorias teóricas mobilizadas no presente estudo, assumimos o caráter introdutório do presente texto, visto como uma problematização inicial sujeita a aprofundamentos futuros (inclusive com a articulação de fontes empíricas) e fomentador de debates reflexivos entre as pares.

Para desnudar os discursos do “amor” que tanto nos violentam na profissão, adotamos o referencial teórico-metodológico do triedro negativo do cuidar, fundamentado no referencial metodológico da hermenêutica de profundidade à análise de ideologias, adaptado as práticas discursivas do cuidado. A versão negativa do triedro do cuidar se baseia na dialética negativa de Theodor Adorno e conforma-se em três momentos: descuidar, confrontar e arrepiar^(17)^. A crítica se inicia com o descuidar, isto é, a negação do que nos aparece imediatamente dado. Descuidamos, então, das concepções de cuidado envoltas em preceitos, dogmas ou romantizações da noção ilusória de “amor”, termo que sintetiza todas as adjetivações correlatas, dantes descritas. Uma vez negado o autodiscurso na enfermagem, confrontamos as versões com seus próprios fundamentos e com a realidade concreta de trabalho da profissão (o que corresponde à crítica imanente da dialética negativa), contrastando o que é dito e como as coisas realmente acontecem no cotidiano de enfermeiras e técnicas de enfermagem. O arrepiar, terceiro momento da desconstrução (adaptado da noção de “estremecer” em Adorno), não fornece respostas fechadas, mas exacerba os incômodos, por vezes físicos, advindos da análise discursiva realizada, ampliando espaços para interpelações e mantendo viva a negatividade da crítica.

O artigo se estrutura em três tópicos. No primeiro, refletimos sobre a contradição entre cuidado e capitalismo segundo Nancy Fraser, reconstruindo sua argumentação sobre o cuidado como trabalho de reprodução social. No segundo, procedemos à crítica imanente do autodiscurso do amor na enfermagem por meio do triedro negativo do cuidar. Na conclusão, recalculamos alternativas para uma rota crítica do trabalho do cuidado no pensamento científico, na formação e na prática política da profissão. Todas as traduções realizadas neste artigo são de nossa responsabilidade.

## CRISE DO CUIDADO EM NANCY FRASER: CONTRADIÇÕES ENTRE CAPITALISMO E TRABALHO DE REPRODUÇÃO SOCIAL

A compreensão do cuidado nas sociedades capitalistas exige deslocá-lo do campo moral, afetivo ou vocacional para situá-lo como trabalho no interior das relações sociais que sustentam a reprodução da vida e a acumulação de capital. Essa reflexão vai além da perspectiva material, propõe a ampliação do capitalismo como uma ordem social institucionalizada que depende de condições não econômicas, como as do trabalho de reprodução social^(5,6)^. Outra distinção necessária corresponde aos conceitos de reprodução social e trabalho do cuidado^(21)^. A reprodução social contempla os processos de manutenção cotidiana e intergeracional que produzem os seres humanos e seus vínculos, os quais incluem a cultura e as instituições sociais. O trabalho do cuidado, por sua vez, corresponde a uma dimensão da reprodução social caracterizada pelas atividades diretamente voltadas à manutenção do bem-estar social das pessoas, tais como cuidar de idosos e crianças, manter lares e sustentar comunidades. Assim, todo trabalho do cuidado integra a reprodução social, mas nem toda reprodução social se reduz ao trabalho do cuidado, posto que inclui outras dimensões, como a educação, a socialização e a organização simbólica da vida. A crítica feminista ao marxismo tem como foco as desigualdades de gênero no capitalismo, visto que o trabalho para manutenção da vida recai sobre as mulheres. O paradoxo questionado consiste nisto: embora as atividades do cuidado sejam indispensáveis à sociedade, o capitalismo as trata como gratuitas, naturais e externas à economia, ocultando a exploração do trabalho^(5,6,21)^.

No âmbito do capitalismo, ocorre uma inversão semântica reducionista entre trabalho e emprego, em que este representa uma pequena parcela daquele, caracterizado pela relação formal e remunerada da produção capitalista. Com isso, as atividades de reprodução social voltadas para atender às necessidades são vistas como improdutivas, por supostamente não gerarem valor econômico no sentido do capital (mercadorias), embora sejam condições prévias para toda e qualquer exploração capitalista. Para Nancy Fraser, essa separação entre a dimensão social (reprodutiva) e econômica (produtiva) foi artificialmente construída pelo capitalismo, perfazendo uma contradição estrutural em que o capitalismo depende do trabalho de reprodução social, mas simultaneamente o invisibiliza, desvaloriza e “canibaliza”. Daí resulta a “crise do cuidado”, que devora a ecologia, o poder político e os laços sociais, coloniza etnias, racializa e gentrifica a reprodução social^(5)^. De fato, desde a consolidação do capitalismo industrial na Inglaterra no século XIX, coincidente com a moral vitoriana, a divisão que associa o trabalho produtivo à produção de mercadorias e o reprodutivo à esfera doméstica ignora que o trabalho de reprodução social produz aquilo que torna possível a exploração no capitalismo, a saber, os seres humanos. A separação entre produção e reprodução também possui um caráter gentrificado, visto que o trabalho do cuidado é considerado “dever” das mulheres nas sociedades patriarcais. Esse processo não apenas naturalizou a responsabilidade das mulheres pelo cuidado, mas institucionalizou novas formas de subordinação: a exploração, a espoliação e a domesticação do trabalho, enovelado na ideologia do “amor”^(5,6)^.

Nesse sentido, a exploração se refere à extração de mais-valor do trabalho assalariado, na qual o trabalhador duplamente livre recebe um salário equivalente ao custo de sua reprodução (ou seja, apenas o suficiente para se manter vivo, em condições de contínua exploração), enquanto o capital se apropria do valor excedente produzido. Ele é considerado “duplamente livre” porque pode vender sua força de trabalho, mas também porque não dispõe dos meios de produção, enredando-se contraditoriamente nas relações de troca. Noutras palavras, o processo ocorre sob a aparência de uma livre troca de equivalentes, mas implica a apropriação sistemática do tempo de trabalho excedente pelo capital. A espoliação, por sua vez, consiste na captura direta e coercitiva de recursos, terras e corpos de trabalho que viabilizam a expansão do capitalismo, incluindo a colonização de países periféricos, como o Brasil. Diferentemente da exploração, que pressupõe o trabalhador formalmente livre, a espoliação opera por meio de violações, confisco bruto e pilhagem, frequentemente associados a processos de racialização, colonialismo e subordinação política. Mais ainda, a espoliação não é um fenômeno em extinção ou restrito às origens do capitalismo, mas uma característica estrutural que reduz os custos da produção para manter a acumulação de capital à custa de populações subjugadas, racializadas e migrantes. Diferentemente do trabalho formalmente explorado, a espoliação não cobre os custos de vida de homens e mulheres racializadas que formam a mão de obra barata, subalterna e precarizada^(6)^.

A terceira dimensão, a domesticação do trabalho, refere-se ao conjunto das atividades do cuidado para a sustentação da vida que historicamente foram sentimentalizadas como amor, inerentes às mulheres, portanto constituídas como trabalho invisível, desvalorizado e frequentemente não remunerado. O termo “domesticado” não se refere ao espaço doméstico, mas expressa um processo histórico de docilização dos corpos e mentes das mulheres. Ele inclui também a naturalização das atividades do cuidado, que passam a ser percebidas como expressões de afeto, obrigação moral ou vocação – e não como trabalho. Obviamente, as três dimensões (exploração, espoliação e domesticação) não agem de forma isolada, mas constituem um sistema integrado de apropriação do trabalho e da vida. Enquanto a exploração extrai valor do trabalho assalariado, a espoliação fornece recursos e reduz custos, ao lado da domesticação que disciplina corpos, pavimentando a usurpação simbólica e material. Nessa perspectiva, o capitalismo não se sustenta apenas pela exploração econômica formal, mas pela apropriação sistemática e domesticada do trabalho de reprodução social, visto como um recurso natural e infinito, embora inscrito na contradição entre capitalismo e cuidado^(5,6)^.

Compreender o cuidado como trabalho de reprodução social permite situá-lo no centro da crise do capitalismo, evidenciando que ele não é um complemento da extração de mais-valia, mas sua condição de possibilidade. O cuidado constitui, portanto, não uma dimensão ética, afetiva, “bondosa” ou relacional da vida social (ou das mulheres), mas um elemento estrutural da economia política, cujo apagamento é funcional à acumulação capitalista. A perspectiva do cuidado como trabalho de reprodução social permite desvendar a desvalorização do trabalho das mulheres na enfermagem, uma vez que as condições históricas dependem da forma como nos inserimos, confrontamos ou reeditamos a ordem social capitalista que “canibaliza” o cuidado. Nesse sentido, vimos que a produção científica da enfermagem, em geral, enaltece uma das principais formas de domesticação do cuidado no capitalismo, maquiando-o como “amor”, com requintes de apelos morais e pitadas do discurso de autoridade da ciência^(15)^. Em contraponto, no próximo tópico descuidamos, confrontamos e arrepiamos a concepção de “amor” que violenta as mulheres na enfermagem.

## DESCUIDAR, CONFRONTAR E ARREPIAR DO “AMOR” NA PROFISSÃO

Descuidemos do que propriamente significa “amor” no autodiscurso da profissão e por que ele se confunde com cuidado. Quais as características o amor assume, o que diz ou esconde? De onde vem a premissa de que o amor é sempre “bondoso”, assim como o cuidado e as enfermeiras que o realizam? O que propriamente significa “bom” e quem impõe a bondade para nós, mulheres? Porque desejamos parecer boas, caridosas, altruístas, heroínas e santificadas, se somos humanas, tornadas “Mulheres”^(23)^, portadoras de um corpo que deseja e sofre, perecíveis e imperfeitas? Por que o discurso do “cuidado como amor”, explicitamente romantizado, atrai parte das mulheres da enfermagem? Do que nos escondemos e por que ele é tão sedutoramente confortável aos nossos ouvidos? Muitas dessas questões não cabem na proposta deste artigo, mas as colocamos como um exercício de descuidado reflexivo. Fiquemos, então, com o principal, o que é dito sobre o “amor” e seus significados na enfermagem.

Tal como acontece com as indefinições sobre o cuidado, o amor não aparece como objeto de reflexão ou aprofundamento teórico na ciência da enfermagem, antes é aludido como “bondade” autoevidente, em que “o amor e o cuidar são dois dados universais”^(24:90)^, de teor dogmático e imune a crítica. Os termos “cuidado”, “amor” e “enfermagem” são permutáveis, tratados como sinônimos de atributos meramente morais, assumidos como uma “ideologia do cuidar”, “um ideal e valor do cuidado […] que terá que se tornar um desejo”. Algumas das imprecisões conceituais são evidenciadas pela expressão “Cáritas” (de origem lexical “caridade”), apresentada como “explícita conexão entre cuidado e amor”^(4)^; ou ainda como “atemporal”; “núcleo da ciência e da enfermagem”; “forças cósmicas mais espantosas e misteriosas”; “essência da enfermagem e o foco mais central e unificador da prática”. Cuidado, enfermagem e amor se entrelaçam de tal maneira, dizem ainda, que “temos que impor ao nosso comportamento, e não ao dos outros, a nossa vontade para cuidar e amar”^(24)^. No mesmo tom impositivo, os “Dez processos Cáritas” sintetizam os mandamentos do cuidado à enfermagem, os quais incluem o “perdão”, os “valores altruístas” da bondade, empatia, preocupação e amor; a aceitação e a “abertura ao milagre”^(4,24)^, vocabulário muito próximo do decálogo catolicista.

O discurso do amor-cuidado-enfermagem gera ilusões de perfectibilidade, de divinização, de ascetismo e de “descorporificação como pretensa superioridade das(os) enfermeiras(os)”, afirma uma das mais incisivas críticas feitas por pesquisador da área^(25)^. De fato, a autoexaltação hiperbólica das(os) enfermeiras(os) corre o risco de cair no desvario. A prática da enfermagem e as agentes são denominadas “um mistério a ser vivido”, que estaria “além do objetivável” e “preocupada com verdades ocultas”. A enfermagem não é um trabalho historicamente situado no tempo e no espaço (portanto sujeito a pressões por mudanças), mas “um conceito filosófico que sugere ternura” e “paixão”, na qual se “oferece a promessa de preservação do ser humano na sociedade”. Os enfermeiros – nunca flexionados na linguagem de gênero – são aqueles que “veem o mundo diferente em relação ao paradigma da ciência médico-natural”, pois cuidam “da totalidade da personalidade humana”. São seres espiritualmente evoluídos, “criativos que se autotranscendem”, os quais devem “considerar algumas ideias metafísicas e morais como guias para seus próprios esforços”. Do enfermeiro se afirma ainda que seria o “único que ouve e honra a história do outro e toda a intensidade dos sentimentos”, pois ele “pode ter acesso indiretamente à mente de uma pessoa e ao seu interior”, portanto estaria “apto para formar uma união com outra pessoa, em um nível que transcende o físico^(4,24)^.

A leitora e o leitor hão de concordar conosco que se trata de uma visão da enfermagem completamente desconectada da realidade do trabalho do cuidado, além de politicamente nociva ao enfrentamento das desigualdades sociais na enfermagem^(10,11,12,13,14,15,16,17,18,19,20)^. Dizer que se trata de um “ideal”, sabidamente abstrato, não exime as defensoras do “amor-cuidado” das consequências opressivas sobre a realidade concreta das enfermeiras e técnicas, a maioria conformada no mito do amor materno/romântico^(5,6,21,23)^. Que a teoria e os processos caritativos do cuidado são inaplicáveis na prática profissional comprova o óbvio^(2,3,25)^, dado o flagrante grau de fantasia das concepções transcritas. As posições críticas dessa vertente atestam que o cuidado-amor é “apenas de um ideal entre outros”, politicamente irrealista, que ignora e fortalece as poderosas estruturas de poder contra nós. As problematizações continuam ao afirmar que esse paradigma do cuidado foi concebido para ser “inefável”, permanentemente alusivo, desejoso de exercer seu poder sobre os outros e inflar a autoestima da profissão^(25)^.

Em concordância com essas contraposições, destacamos o olhar do cuidado como trabalho de reprodução social, tomado aqui como expressão do descuidado crítico-discursivo das falácias do amor mitificado nas relações capitalistas. Assumimos a interpretação feminista do amor como ideologia mitificada que violenta simbolicamente as mulheres e fortalece a dominação masculina^(5,6,21)^. Sob o patriarcado, o amor como investimento libidinal é estruturado para docilizar corpos femininos, moldando-os para permanecer subjugado como “segundo sexo” (objeto), em que o “primeiro” pertence ao universo masculino (sujeito)^(23)^. Nesse entendimento, diante da disparidade entre o pretensioso “amor-cuidado” e as indignas condições de trabalho de enfermeiras e técnicas que atuam na assistência, caberia questionar como os ideais de pureza e perfeição se introjetaram tão fortemente na profissão. Na contextualização sócio-histórica do “capitalismo canibal” realizada por Nancy Fraser, encontramos uma pista importante. Há três fatos históricos na consolidação do capitalismo no século XIX que se entrelaçam nefastamente na profissionalização da enfermagem moderna, a saber: i – o fortalecimento do capitalismo industrial e da Inglaterra como centro de poder político e econômico; ii – a moralidade da era vitoriana; iii – o movimento feminista inglês contra a lei de doenças contagiosas. Expliquemos melhor como essas pontas se encontram na enfermagem profissional.

A começar pelo primeiro ponto, o fortalecimento da era industrial do capitalismo ratifica a separação entre o trabalho produtivo (econômico, masculino) e o reprodutivo (social, feminino), construído graças à colonização e domesticação das mulheres como “donas de casa” e “anjos do lar”. Nisso, o esforço para garantir a reprodução social restrita ao trabalho não pago se entrelaçou vigorosamente com a dominação masculina. Na Inglaterra, eis o segundo ponto, o capitalismo e a dominação masculina coincidem com o puritanismo da era vitoriana e o surgimento do modelo nightingaliano da enfermagem, cujo poder se espalha mundo afora. Nesse contexto de austeridade moral, o enraizamento do capitalismo industrial ocorreu diferente para as mulheres pobres em relação às ricas, com insatisfação de ambos os lados. As mulheres pobres racializadas não atendiam aos ideais vitorianos de domesticidade (recato, submissão ao marido ou pai, restrição às tarefas domésticas ou atividades tidas como femininas, regulação do corpo e comportamento social). Por outro lado, as mulheres ricas, como Florence Nightingale, usufruíam de conforto material e prestígio moral, mas clamavam por liberdade e autonomia jurídica. Segundo Nancy Fraser, a “solução” da separação entre trabalho produtivo e reprodutivo se deu às custas das mulheres, colocando-as umas contra as outras. O conflito consistia nisto: as preocupações filantrópicas das mulheres ricas/médias da era vitoriana divergiam dos interesses materiais das “companheiras decaídas”, acentuadas por julgamentos morais em relação ao exercício da prostituição^(5)^.

O ápice do enfrentamento moral entre as mulheres na era vitoriana culminou com a luta pela revogação da Lei de Doenças Contagiosas (*Contagious Diseases Acts*), capitaneada pelo movimento “Revolta das Mulheres” (1864 a 1869), terceiro ponto à historicidade da enfermagem^(26)^. A mencionada lei visava controlar as infecções sexualmente transmissíveis entre os militares, e para isso submetia as mulheres prostitutas a exames ginecológicos forçados e internação compulsória. Curiosamente, mas por razões opostas, a afrontosa lei uniu as mulheres de distintas classes, capitaneadas pelo movimento feminista da Associação Nacional das Senhoras (*Ladies National Association*), que denunciava as leis como discriminatórias e violentas. As trabalhadoras do sexo, principais atingidas, rebelaram-se igualmente pelas agressões sofridas. Algumas senhoras da aristocracia se juntaram ao movimento por razões filantrópicas; outras, como Florence Nightingale, por razões morais, sob o argumento que a lei “sancionava oficialmente o vício” da prostituição. Embora a lei tenha sido revogada, os desdobramentos desse acontecimento ratificaram as normas de pureza social e deram novos poderes policiais de vigilância sobre a classe operária^(26)^.

Mas como a profissionalização da enfermagem moderna é influenciada por contextos históricos aparentemente tão distintos? A nosso ver, a ascensão do capitalismo e o poder da classe aristocrata, o puritanismo, os valores de branquitude e pureza social dela advindos, foram incorporados ao modelo nightingaliano, no qual se atribui supostamente às *lady nurses* (enfermeiras) um *status* de superioridade moral em relação às *nurses* (técnicas de enfermagem), com subjugação das mulheres e vigilância de corpos. Que esse modelo moral de enfermeira padrão influenciou a formação no mundo inteiro, inclusive em países colonizados, racializados e brutalmente espoliados como o Brasil, é bem discutido pela literatura^(7,8,9)^. A saída moralizante e cientificista do modelo nightingaliano não conseguiu tamponar as contradições das mulheres reais, com corpo, raça e classe social, sobrando iniquidades e opressões no trabalho, fora e dentro da equipe da enfermagem^(10,11,12,13)^. No entanto, o disciplinamento das mulheres que perfazem 80% da força de trabalho em saúde precisou de um ingrediente a mais, o mais poderoso, consubstanciado na alcunha do “anjo branco”, a face mítica da “enfermagem-cuidado-amor”. Ora, a falaciosa candura abnegada da enfermagem nada mais é do que a colonização das mulheres pela ideologia do “anjo do lar” transposta para a o hospital, naturalizando a separação entre produção e reprodução capitalista^(5,7,8)^.

Face à historicidade descrita, verificamos que a noção de “cuidado-amor” ainda prevalece no discurso científico, na formação e na prática da enfermagem, resultando em fragilidade política e iniquidades. Uma das evidências do autorismo moral da profissão transparece na afirmação de que “temos que impor ao nosso comportamento […] a nossa vontade para cuidar e amar”^(24:62)^, retrato fiel da domesticação do trabalho da enfermagem^(5,6)^. Não raro o cuidado da enfermagem é enaltecido como “obrigação ou dever”, quando se trata de espoliação do trabalho emocional, entendido como o esforço de gerenciar, suprimir ou expressar emoções específicas exigidas no desempenho de uma função, visando atender às expectativas normativas. O trabalho emocional perfaz uma das formas de opressão do trabalho de reprodução social^(27)^. Além das explorações emocionais no trabalho formal, a prescrição do cuidado-amor expressa o grau de rigidez moral endógeno à enfermagem. A esse respeito, pesquisas apontam a alta prevalência da síndrome de *burnout* em enfermeiras e técnicas, bem como os esgotamentos decorrentes dos trabalhos emocionais da profissão. No entanto, em geral, o trabalho emocional e de reprodução social não são problematizados pelas cientistas da enfermagem, mas vistos como atributos da profissão, o que renova a força opressiva do “amor”^(11,28)^.

Uma vez descuidadas discursivamente as falácias do “amor” na enfermagem, que contrastam com a dura realidade de trabalho da profissão, confrontemos o autodiscurso com suas bases teóricas. Problematizemos primeiro a inespecífica e controversa concepção “metafísica” do cuidado, no máximo descrita como uma dimensão espiritual que estaria “fora do tempo”^(1,4,24)^. Trata-se de uma simplificação grosseira, supostamente referenciada na noção de *Dasen* (aquele que possui a capacidade de questionar o sentido do ser) de Heidegger. Por limitação de espaço e propósito, não cabe alinhavar toda a discussão por detrás de conceitos filosóficos profundos, estaríamos atuando no mesmo grau de superficialidade que contestamos. Pedimos licença, entretanto, para delimitar o centro da controvérsia. A metafísica é uma tradição filosófica referente à busca dos universais da verdade, passando por várias guinadas e críticas, a principal efetuada por Heidegger. Seu questionamento consiste no esquecimento da pergunta pelo ser (algo que subsiste por seu sentido, incapturável por conceito) pela metafísica tradicional, que passa a se ocupar dos entes (o que falamos, o que entendemos, interpretamos; é o que é, como nós somos). Na visão heideggeriana, ao inquirir pelo “ser” do ente, por meio da noção de *Dasein* (ser humano) que se abre para essa revelação, a filosofia superaria a metafísica clássica, significando nisso uma intervenção crítica, enquanto “destruição” da tradição, especialmente pela inovadora compreensão da dimensão temporal do ser. Assim, a afirmação de que o cuidado seria “atemporal” contradiz frontalmente a base fundante do *Dasen*, pois o ser só existe no tempo (daí o nome “Ser e Tempo”)*,* além de situar a enfermagem fora da história, incapaz de mudar seu destino^(29)^.

O segundo confronto diz respeito à pretensa representação “da herança e dos fundamentos de Nightingale”^(4)^ na noção sentimental de cuidado como “bondade”, “amor” “ternura”, “compaixão” e correlatos. A despeito do puritanismo moral e protestantismo que tanto influenciaram o conservadorismo da profissão, bem sabemos que Florence Nightingale defendia uma ciência racional e empírica para o cuidado da enfermagem, sendo avessa a sentimentalismos fáceis. Dessa forma, alguns de seus posicionamentos contestam frontalmente a noção sentimental atribuída ao cuidado-amor, por exemplo: “Não é tarefa da enfermagem ser bondosa, no sentido de fazer a própria vontade ou satisfazer os próprios sentimentos; é sua tarefa cumprir as leis da saúde”; ou ainda: “uma enfermeira não é uma mulher devotada e obediente, mas deve ser uma pessoa reflexiva e instruída”^(30:200)^. Com isso, a pretensa representação dos pensamentos de Florence na noção de cuidado como amor cai por terra por contradizer seus alicerces.

Uma vez descuidado e confrontado o autodiscurso do amor, chegamos ao arrepio, momento em que nos permitimos abstrações, incômodos, interpelações, críticas e autocríticas. Retomemos as perguntas iniciais, especialmente o motivo de o discurso romantizado do “amor” atrair parte das mulheres da enfermagem, ou por que ele é tão sedutoramente confortável aos nossos ouvidos. Quiçá a dor recôndita que afagamos com autoenganos seja a mesma que nos une na condição de “Mulher”, como o “Outro” do “Eu” universal, renegada à condição de objeto nominado pela autoridade do sujeito masculino. No livro II do *Segundo Sexo*, Simone de Beauvoir discute as experiências amorosas da mulher, o que ela faz da sua condição, como sente e que evasões lhe são permitidas. A partir dos entrelaçamentos das categorias da “narcisista” (amor ao eu é um fim absoluto), “apaixonada” (mitificação do amor romântico) e “mística” (entrega e devoção do amor à Deus), a filósofa reflete sobre as circunstâncias que levam as mulheres a se voltarem para si e dedicarem o seu amor, como uma espécie de compensação do que lhe fora negado como sujeito^(23)^.

Em consonância com as reflexões de Beauvoir, parece que a necessidade de autoafirmação, superioridade moral e identidade imutável expressa nos discursos da enfermagem se entrecruza com a dor latente do “Outro” que pertence a todas nós, mulheres condicionadas pelo tempo e pelo que fazemos nele. No mais, “esses esforços de salvação só podem redundar em fracassos; ou a mulher põe-se em relação com um irreal; seu duplo ou Deus; ou cria uma relação irreal com um ser real”^(23:876)^.

## CONSIDERAÇÕES FINAIS

Precisamos falar sobre o cuidado como trabalho de reprodução social na enfermagem, incluir a perspectiva de gênero e o diálogo fecundo com a epistemologia feminista nos currículos da graduação e da pós-graduação, com repercussões nas produções científicas e na prática política da profissão. Ao invés da busca improvável por “universais” de verdade ou de mistérios exotéricos que não contribuem com a práxis ou com o perfil crítico registrado nos projetos pedagógicos de cursos, ampliemos a visibilidade da resistência crítica e autocrítica da produção científica de enfermeiras pesquisadoras. Com isso, desvelamos os discursos totalitários do “amor” que aprisionam nosso agir reflexivo e nossa capacidade de construir a história da profissão. A ciência que produzimos há que problematizar as contradições sociais e de gênero na enfermagem, cujo trabalho tem sido rigidamente domesticado nos bancos formativos de enfermeiras e técnicas de enfermagem. Contestemos igualmente a pretensa neutralidade científica enovelada com a busca autocentrada de evidências como discurso de autoridade, desvelando seu comprometimento ideológico com a ordem social capitalista machista, misógina, sexista, racista e antiecológica que nos canibaliza sob falsas cantilenas de nobreza. O conhecimento que produzimos não pode se eximir da dimensão política e do comprometimento com a melhoria das condições de trabalho, a valorização salarial e o enfrentamento das violências na profissão, dentre outras pautas inadiáveis.

Para uma rota crítica do cuidado na enfermagem, percebamos, enfim, que a nossa riqueza consiste justamente no que fora sistematicamente apagado pelo conservadorismo e elitismo da enfermagem moderna: a diversidade de culturas, gêneros, sexualidades, raças, classes e saberes das mulheres que trabalham arduamente pela saúde da população brasileira, nos distintos espaços da reprodução social, não apenas na assistência. Quem sabe com isso possamos inaugurar outras formas de amor, mais próximo da dimensão política e econômica condizente com a democracia das mulheres.

## Data Availability

Todo o conjunto de dados que dá suporte aos resultados deste estudo foi publicado no próprio artigo.

# Translation: No es amor, sino trabajo de reproducción social: el cuidado y la enfermería a la luz de Nancy Fraser

**DOI:** 10.1590/1980-220X-REEUSP-2026-0127es

**Authors:** Maria Raquel Gomes Maia Pires, Gabriela Duarte Almeida Mundim

**Affiliations:** 1 Universidade de Brasília, Faculdade de Ciências da Saúde, Departamento de Enfermagem, Brasília, DF, Brasil.; 2 Universidade de Brasília, Centro de Estudos Avançados Multidisciplinares, Brasília, DF, Brasil.

**Keywords:** Estereotipo de género, Enfermería, Cuidado de enfermería, Discurso, Feminismo, Trabajo

## Abstract

Estudio teórico que adopta la concepción del cuidado como trabajo de reproducción social de Nancy Fraser como crítica al autodiscurso científico del cuidado como “amor” en la enfermería. Argumentamos que el cuidado como trabajo de reproducción social —una acción esforzada que busca satisfacer las necesidades sociales de la fuerza laboral en las sociedades capitalistas— pone de manifiesto las fragilidades del “amor” en la enfermería, al revelar las condiciones materiales, interseccionales, capitalistas y sociohistóricas que lo sustentan, lo que sustenta prácticas contrahegemónicas. Inicialmente reflexionamos sobre la contradicción entre el cuidado y el capitalismo. A continuación, procedemos a una crítica inmanente del autodiscurso del amor en la enfermería a través del triángulo negativo del cuidado. En la conclusión, replanteamos alternativas para una trayectoria crítica del trabajo de cuidado en la profesión. Proponemos la inclusión de la perspectiva de género y el diálogo con la epistemología feminista en los planes de estudio de pregrado y posgrado, con repercusiones en la producción científica y en la práctica política de la enfermería. La ciencia que producimos debe cuestionar las contradicciones sociales y de género en la enfermería, cuyo trabajo ha sido rigurosamente domesticado.

## INTRODUCCIÓN

La concepción del cuidado en el discurso científico de la enfermería, ya sea en la formación, en la práctica o en las organizaciones profesionales en general, se ha confundido históricamente con un deber o un atributo moral, basado en nociones erróneas de bondad, amor, altruismo y benevolencias afines que supuestamente elevarían la nobleza de la profesión. Existe un autodiscurso por parte de las enfermeras investigadoras que posicionan el cuidado como “práctica amorosa”, “compasión”, “la esencia y el núcleo disciplinario” de la profesión, a veces como si fueran términos intercambiables, es decir, como si el cuidado y la enfermería fueran sinónimos^(1,2,3,4)^. De manera más evidente, las teóricas de la enfermería evocan “explícitamente el amor y el cuidado, buscando la fusión hacia un paradigma ampliado que se conecte con las dimensiones espirituales y los procesos vitales de las experiencias humanas”^(4:132)^.

En contraposición a esta corriente teórica que se autodenomina representante “[…] de la herencia y los fundamentos de Nightingale”^(4)^, concebimos el cuidado en la materialidad de las relaciones sociales de producción, es decir, como trabajo de reproducción social en el sentido de Nancy Fraser, entendido como un conjunto de acciones esforzadas que tienen como objetivo satisfacer las necesidades sociales de la fuerza de trabajo (de alimentación, refugio, vivienda limpia, descanso, bienestar, la salud, la educación y otras) en las sociedades capitalistas. El trabajo reproductivo se caracteriza por condiciones interseccionales que explotan, despojan y domestican (disciplinan) injustamente el trabajo de las mujeres, incluyendo a las enfermeras, pero también a otras que asumen las tareas domésticas, cuidan a los hijos, a los adultos mayores y similares^(5,6)^. La comprensión del cuidado como trabajo de reproducción social del capitalismo no se limita a la dimensión asistencial de la enfermería, sino que abarca todas las acciones pertinentes a la práctica de las enfermeras y técnicas en el ámbito de las políticas públicas de salud y educación (gestión, educación, investigación, asistencia), en un sentido amplio, como proceso de reproducción material y simbólica de la fuerza laboral en el capitalismo. En este estudio, utilizamos esta concepción del cuidado como crítica al discurso ilusorio de la profesión, es decir, aquel que concibe el cuidado como “amor” y adjetivos correlatos, descritos brevemente más arriba y analizados en los párrafos siguientes.

Para empezar, en sintonía con las reflexiones de la epistemología feminista sobre las violencias interseccionales de género, raza, clase, sexualidad y edad que nos afectan a nosotras, las mujeres, sean enfermeras o no, nos preguntamos si todas las personas que realizan el trabajo de cuidado son (o deben ser) enfermeras, como parecen sugerir las teóricas de la enfermería^(1,2,3,4)^. Pensemos juntas: ¿qué gana la profesión al asumir heroicamente, como una controvertida “esencia” inmutable^(1,4)^, todas las violencias simbólicas, institucionales y estructurales implicadas en la responsabilidad desigual que se le atribuye a las mujeres por el trabajo de cuidado? ¿No sería políticamente más estratégico si hiciéramos justo lo contrario, es decir, si enfrentáramos desde dentro la falacia opresiva de los enunciados que solo domestican los cuerpos y nublan el pensamiento crítico de las mujeres en la enfermería? Sin duda, esta es solo una de las cuestiones, pero hay mucho más en juego cuando reeditamos de manera hegemónica los discursos ideológicos del “amor” en la enfermería. Expliquémoslo mejor.

El discurso de la supuesta “bondad amorosa” del cuidado impide que se revelen y se enfrenten colectivamente las contradicciones arraigadas en la profesión, entre ellas: i – las fragilidades epistémicas sobre el cuidado y los esfuerzos insostenibles por convertirlo en un “ideal moral” o una “ciencia unitaria” de “amor infinito”, inmune a la crítica^(1,2,3,4)^; ii – la nefasta influencia de la blancura, del origen eugenésico, racista y clasista de la profesionalización de la enfermería, generadora de prácticas discursivas que ratifican endógenamente las opresiones contra nosotras, las mujeres^(7,8,9)^; iii – la sobreexplotación del trabajo de enfermería en el contexto capitalista, los bajos salarios y las numerosas enfermedades que padecen las enfermeras y el equipo, a veces naturalizadas como parte del deber del oficio, altruismo o supuestos “heroísmos”^(10,11,12,13,14)^; iv – la violencia simbólica de los discursos científicos estereotipados y moralizantes, perpetrados acríticamente en la formación y en la práctica política de la profesión^(15)^; v – la visión errónea de que sería “la sociedad” la que estigmatizaría y menospreciaría la profesión, como si la enfermería no formara parte ni contribuyera a la forma en que se nos retrata^(16)^.

Una mirada atenta a la influencia del “proceso cáritas”^(1,4)^ en la enfermería pone de manifiesto fácilmente que la sociedad nos ve exactamente como insistimos hegemónicamente en presentarnos (o negarnos), como parte de ella que somos. Nos referimos aquí a los cómodos argumentos de que es la sociedad la que menosprecia y impone estereotipos de género contra la profesión, como si estuviéramos fuera de ella^(14,16)^. Sin embargo, si el discurso predominante y contradictorio de buena parte de las enfermeras científicas insiste en afirmar que somos seres moralmente superiores, dueñas del cuidado como una esencia “unitaria” de la profesión, altruistas y heroínas (siempre blancas)^(14)^ en busca de un ideal de perfeccionamiento, cuando la desigualdad social, el origen racializado de las mujeres que siempre han cuidado y el trabajo concreto de la profesión evidencian claramente lo contrario, ¿por qué esperar que “los demás” nos vean de manera diferente? Mientras camuflamos la dura realidad laboral de la profesión con proyecciones de deseos de pureza y estereotipos de género^(15)^, abundan la precariedad, la desvalorización, la invisibilidad y la violencia de todo tipo en la realidad del trabajo de reproducción social de las enfermeras y las técnicas de enfermería^(10,11,12,13)^.

Además, mientras que parte de las enfermeras investigadoras se ocupan de moralizaciones que recaen de manera opresiva sobre la profesión, investigadoras e investigadores de otras áreas realizan críticas precisas sobre las herencias ideológicas, eugenésicas, elitistas y racistas de la profesionalización de la enfermería^(7,8)^, lo que sin duda contribuye al carácter de “superexplotación” del trabajo en el contexto neoliberal del Sistema Único de Salud^(13)^. Ya es hora, pues, de que escuchemos atentamente a “la sociedad”, representada ahora en la crítica aguda que otros investigadores hacen de nosotros, en lugar de escondernos tras análisis infundados. Nos corresponde a nosotras, las enfermeras, construir la agencia de lucha política de nuestra historia, lo cual incluye la crítica y la autocrítica de la ciencia que producimos. Este estudio teórico se inserta precisamente en esa brecha de producción reflexiva sobre el cuidado y la enfermería.

En cuanto a las demás dificultades de la profesión mencionadas anteriormente, comentemos las incongruencias ocultas tras el frágil “velo” del cuidado amoroso, contra las cuales nos oponemos junto con Nancy Fraser^(5)^. Empezando por la reconocida dificultad para conceptualizar el cuidado desde la ciencia de la profesión, una revisión bibliográfica de 39 estudios identificó algunos atributos del cuidado en la práctica de la enfermería, caracterizados genéricamente como: “comunicación terapéutica”; “presencia junto a la cama del paciente”; “empatía”; “cuidados científicos”; “subjetividad”; “ayuda al bienestar”; e incluso la jerga de “ciencia, arte y creatividad”. En otros intentos de mapeo, también figuran el “ideal moral”, la “práctica ética”, la “experiencia existencial”, la “práctica de la bondad amorosa” o la “práctica espiritual”^(3)^. Evidentemente, gran parte de estas publicaciones se inspiran en la Teoría del Cuidado Humano de la enfermería, revisada recientemente^(1,4)^. Un estudio cuantitativo identificó 79 trabajos sobre esta teoría, correspondientes a 221 autoras y autores y 155 instituciones de 18 países, distribuidos en todos los continentes, lo que atestigua la fuerza preponderante del cuidado como “amor” en el discurso científico de la profesión^(2)^.

Paradójicamente, en ninguna de las publicaciones revisadas^(2,3)^ se conceptualiza el cuidado en el contexto socioeconómico de las relaciones sociales concretas entre seres humanos, ya sea como una acción de ayuda tensionada por la micropolítica de las relaciones de poder^(17)^, ya sea en la esfera del trabajo de reproducción social —que es lo que nos interesa aquí—, lo que significa que las actividades materiales del cuidado, realizadas de manera desigual por las mujeres en la enfermería, quedan al margen de las preocupaciones del pensamiento hegemónico de la enfermería. Ciertamente, si hay poder, hay resistencia; por lo tanto, la corriente teórica que mitifica el “amor” dista mucho de representar el pensamiento único en el discurso científico de las enfermeras; es simplemente la más idealizada, sentimental y perjudicial para la acción política de la profesión. Al respecto, identificamos críticas potentes, relevantes y consistentes en la producción científica de la enfermería que cuestionan estas versiones, tanto a nivel mundial como nacional, con un sólido anclaje en la economía política feminista (Teoría de la Reproducción Social - TRS) y en el pensamiento feminista negro, por citar algunos, con los que dialogamos^(12,17,18,19,20)^.

En torno a este fructífero entrelazamiento de la enfermería con la epistemología feminista, la Teoría de la Reproducción Social —una relectura de la concepción del trabajo en Marx— ha renovado el interés de las teóricas en el campo, ante la crisis estructural del neoliberalismo. Desde la interpretación feminista, el énfasis de la crítica recae en la insostenible división impuesta por el capital entre el trabajo productivo (económico), es decir, aquel que genera valor mediante la producción de mercancías, y el trabajo reproductivo (social), el cual produce y sostiene las condiciones sociales para la reposición de la fuerza de trabajo. En otras palabras, la reproducción de la trabajadora incluye tanto la dimensión biológica como la satisfacción de las necesidades vitales, así como las instituciones, los símbolos y las estructuras de poder de la sociedad, en el sentido amplio de la reproducción social^(21)^.

Existe un debate fructífero entre las feministas sobre si el trabajo reproductivo genera o no valor económico (de intercambio), dado el carácter de mercancía de la propia trabajadora reproducida mediante el cuidado. La pregunta es la siguiente: ¿el producto generado por el trabajo de cuidado, es decir, la fuerza de trabajo humana, es una mercancía con valor que genera plusvalía (trabajo abstracto) en el proceso de intercambio? Las respuestas se dividen en dos corrientes, a saber: i) las obreristas italianas afirman que el valor también se produce fuera de los lugares de trabajo, es decir, en el ámbito privado doméstico (las llamadas “fábricas sociales globales”), por lo que contiene una plusvalía que se incorpora socialmente a la fuerza de trabajo humana. Con ello, el trabajador y el capitalista establecen una relación contractual que ya incluye la plusvalía generada por las mujeres, aunque no sea remunerada; ii) por su parte, la tradición anglosajona, de la que forma parte Nancy Fraser, contraargumenta al afirmar que el trabajo reproductivo no produce valor de cambio, sino algo fundamental: las condiciones que hacen posible toda extracción de plusvalía. En esta segunda corriente, el valor es la expresión de una relación social producida en el contexto del trabajo organizado en términos capitalistas (industrializado, especializado, fragmentado, alienado); por lo tanto, el trabajo de cuidado (como el doméstico) produce valores de uso que se consumen para la reproducción de la fuerza de trabajo, pero no generan plusvalía, en sentido productivo. La diferencia radica en la interpretación de la categoría de valor, algo controvertido dentro del propio marxismo, cuyo análisis en profundidad excede los objetivos de este artículo^(21)^.

Mientras tanto, sea cual sea la interpretación feminista de la teoría del valor en Marx, ambas cuestionan la condición injusta del trabajo de cuidado realizado por las mujeres en las relaciones patriarcales, sexistas y racializadas del capitalismo, que sí es el objeto prioritario de reflexión del estudio. Para la ciencia de la enfermería, la discusión también es importante, dadas las importantes contribuciones críticas sobre la teoría del valor-trabajo en Marx, que surgieron en la década de los ochenta del siglo XX (y que recientemente han cobrado mayor relevancia), un momento singular de la enfermería brasileña en el que coinciden la reflexión y la práctica política sobre la historicidad de la profesión. A nuestro ver, aunque las reflexiones sobre la categoría del valor-trabajo en enfermería no abordan suficientemente las cuestiones de género en la profesión, el diálogo con la epistemología feminista puede fomentar debates fructíferos y tensiones sobre el trabajo de enfermería, ya sea en la dimensión de la reproducción social o en los complejos productivos de la salud, como fuerza laboral superexplotada^(11,12,13,22)^.

Volviendo al contexto de la TRS, la reflexión sobre el trabajo a partir de la relectura de Marx constituye el núcleo de la contradicción identificada por Fraser^(5)^, a saber: el cuidado, cuyo trabajo mantiene las condiciones sociales para que las personas puedan ser explotadas a expensas del capital, es subyugado por el capitalismo, que depende intrínsecamente de ese mismo trabajo de cuidado realizado por las mujeres, sean enfermeras o no. Así, al mismo tiempo que el capitalismo depende visceralmente del cuidado, de manera contradictoria lo devalúa, lo precariza, lo “canibaliza”, lo despoja o no paga por el trabajo reproductivo, sobre todo cuando está históricamente impregnado por la raza y la clase social^(5,6,21)^.

Ante la realidad estructural, material, sociohistórica y cultural del capitalismo, la concepción “amorosa” del cuidado, respaldada por científicos que afirman ser portadores de la herencia histórica de la profesión^(1,2,3,4)^, no hace más que reforzar la “domesticación” (disciplinamiento) de las mujeres para una doble o triple explotación capitalista.

En este sentido, la elección de la TRS de Nancy Fraser como marco analítico se justifica porque con ella podemos desentrañar las múltiples facetas de la explotación del trabajo de enfermería en el ámbito de las relaciones capitalistas, tanto en su faceta más visible, a través del salario, como en las capas invisibilizadas por la explotación no remunerada o por la domesticación simbólicamente idealizada del trabajo de las mujeres. Expliquemos cómo ocurre esto en la enfermería. En la primera dimensión, la explotación se da a través del empleo formal de enfermeras y técnicas de enfermería en las instituciones, el cual es mal remunerado, precario, sobreexplotado, subvalorizado y generador de enfermedades^(10,12,13,14)^. La segunda y tercera usurpación se dan en forma de trabajo de cuidado no remunerado que realizamos nosotras, las mujeres, en los hogares, invisibilizado por la ideología sentimentalizada de que las mujeres “nacieron para cuidar”^(15)^. En cuanto a estas formas de explotación presentes en la esfera pública, un estudio reciente identifica la expoliación (robo) del tiempo de trabajo de las mujeres negras en enfermería, ya que trabajan 26 horas y 8 minutos más que los hombres blancos y 21 horas y 20 minutos más que las mujeres blancas de la profesión, a pesar de que reciben los mismos salarios mensuales^(12)^.

Constatamos, pues, que nos convertimos en presa fácil de la domesticación del trabajo reproductivo por parte del capitalismo, cada vez que ratificamos los discursos que idealizan el cuidado, tratándolo no como un trabajo abstracto y concreto, insertado históricamente en las relaciones sociales de producción y reproducción, y, por lo tanto, modificable mediante la lucha política de la categoría, sino como un “deber moral”, una obligación ética, “una experiencia existencial”, “una esencia” inmutable, “una compasión y una práctica amorosa”, anclado en un supuesto instinto maternal de las mujeres, reeditado por parte de la ciencia y que repercute en la práctica de la enfermería^(1,2,3,4,7,8,9,15)^.

Ante la influencia de los discursos sobre el “amor” en la fragilidad política y económica de la profesión, el estudio plantea la siguiente pregunta: ¿cuáles son las contribuciones de la concepción del cuidado como trabajo de reproducción social, según Nancy Fraser, para enfrentar el autodiscurso del “amor” en la enfermería? Sostenemos que el cuidado como trabajo de reproducción social, entendido como una acción esforzada que busca satisfacer las necesidades sociales de la fuerza laboral en las sociedades capitalistas, pone de manifiesto las fragilidades del autodiscurso del “amor” en la enfermería, al desnudar las condiciones materiales, interseccionales, capitalistas y sociohistóricas que lo sustentan, lo que respalda prácticas discursivas contrahegemónicas en la profesión. El objetivo es analizar críticamente las contribuciones de la concepción del cuidado como reproducción social, según Nancy Fraser, para confrontar el autodiscurso del “amor” en la enfermería.

## MÉTODO

Se trata de un estudio teórico basado en la TRS, según Nancy Fraser, representante de la Teoría Crítica desde una perspectiva feminista, para desvelar la cara opresiva del autodiscurso del cuidado como “amor” en la ciencia de la enfermería.

Adoptamos principalmente las reflexiones sobre las contradicciones entre el cuidado y el capitalismo realizadas por la pensadora^(5,6)^, ya que aportan una comprensión contextual de las relaciones de cuidado, así como para cuestionar las prácticas discursivas que conforman la formación y la práctica de las mujeres en enfermería. La investigación teórica sobre la TRS forma parte de un proyecto de investigación que analiza el cuidado desde la epistemología feminista, lo cual respalda la oferta de asignaturas en los niveles de pregrado y posgrado, así como la orientación de subproyectos sobre cuestiones de género en enfermería. Como limitación, dado el espacio reducido y la cantidad de categorías teóricas utilizadas en el presente estudio, reconocemos el carácter introductorio de este texto, visto como una problematización inicial sujeta a profundizaciones futuras (incluso con la integración de fuentes empíricas) y que fomente debates reflexivos entre colegas.

Para desnudar los discursos del “amor” que tanto nos violan en la profesión, adoptamos el marco teórico-metodológico del triángulo negativo del cuidado, fundamentado en el marco metodológico de la hermenéutica de profundidad para el análisis de ideologías, adaptado a las prácticas discursivas del cuidado. La versión negativa del triángulo del cuidado se basa en la dialéctica negativa de Theodor Adorno y se conforma en tres momentos: descuidar, confrontar y estremecer^(17)^. La crítica comienza con el descuidar, es decir, la negación de lo que se nos presenta como un dato inmediato. Descuidamos, entonces, las concepciones del cuidado envueltas en preceitos, dogmas o idealizaciones de la noción ilusoria del “amor”, término que sintetiza todos los adjetivos correlatos, descritos anteriormente. Una vez negado el autodiscurso en enfermería, confrontamos las versiones con sus propios fundamentos y con la realidad concreta del trabajo de la profesión (lo que corresponde a la crítica inmanente de la dialéctica negativa), contrastando lo que se dice y cómo suceden realmente las cosas en el día a día de las enfermeras y las técnicas de enfermería. El estremecer, tercer momento de la deconstrucción (adaptado de la noción de “estremecimiento” en Adorno), no ofrece respuestas definitivas, sino que exacerba las incomodidades, a veces físicas, derivadas del análisis discursivo realizado, ampliando los espacios para las interpelaciones y manteniendo viva la negatividad de la crítica.

El artículo se estructura en tres temas. En el primero, reflexionamos sobre la contradicción entre el cuidado y el capitalismo según Nancy Fraser, reconstruyendo su argumentación sobre el cuidado como trabajo de reproducción social. En el segundo, procedemos a la crítica inmanente del autodiscurso del amor en la enfermería a través del triángulo negativo del cuidado. En la conclusión, replanteamos alternativas para una ruta crítica del trabajo de cuidado en el pensamiento científico, en la formación y en la práctica política de la profesión. Todas las traducciones realizadas en este artículo son de nuestra responsabilidad.

## LA CRISIS DEL CUIDADO EN NANCY FRASER: CONTRADICCIONES ENTRE EL CAPITALISMO Y EL TRABAJO DE REPRODUCCIÓN SOCIAL

La comprensión del cuidado en las sociedades capitalistas exige sacarlo del ámbito moral, afectivo o vocacional para situarlo como trabajo en el seno de las relaciones sociales que sustentan la reproducción de la vida y la acumulación de capital. Esta reflexión va más allá de la perspectiva material; propone ampliar la concepción del capitalismo como un orden social institucionalizado que depende de condiciones no económicas, como las del trabajo de reproducción social^(5,6)^. Otra distinción necesaria corresponde a los conceptos de reproducción social y trabajo de cuidado^(21)^. La reproducción social abarca los procesos de mantenimiento cotidiano e intergeneracional que dan forma a los seres humanos y sus vínculos, los cuales incluyen la cultura y las instituciones sociales. El trabajo de cuidado, por su parte, corresponde a una dimensión de la reproducción social caracterizada por las actividades directamente orientadas al mantenimiento del bienestar social de las personas, tales como cuidar a personas mayores y niños, mantener los hogares y sostener las comunidades. Así, todo trabajo de cuidado forma parte de la reproducción social, pero no toda la reproducción social se reduce al trabajo de cuidado, ya que incluye otras dimensiones, como la educación, la socialización y la organización simbólica de la vida. La crítica feminista al marxismo se centra en las desigualdades de género en el capitalismo, dado que el trabajo para el mantenimiento de la vida recae sobre las mujeres. La paradoja que se cuestiona consiste en lo siguiente: aunque las actividades de cuidado son indispensables para la sociedad, el capitalismo las trata como gratuitas, naturales y ajenas a la economía, ocultando la explotación del trabajo^(5,6,21)^.

En el marco del capitalismo, se produce una inversión semántica reduccionista entre trabajo y empleo, en la que este último representa una pequeña parte de aquel, caracterizado por la relación formal y remunerada de la producción capitalista. Con ello, las actividades de reproducción social orientadas a satisfacer las necesidades se consideran improductivas, por no generar supuestamente valor económico en el sentido del capital (mercancías), aunque son condiciones previas para toda forma de explotación capitalista. Para Nancy Fraser, esta separación entre la dimensión social (reproductiva) y la económica (productiva) fue construida artificialmente por el capitalismo, lo que constituye una contradicción estructural en la que el capitalismo depende del trabajo de reproducción social, pero al mismo tiempo lo invisibiliza, lo devalúa y lo “canibaliza”. De ahí surge la “crisis del cuidado”, que devora la ecología, el poder político y los lazos sociales, coloniza a los grupos étnicos, racializa y gentrifica la reproducción social^(5)^. De hecho, desde la consolidación del capitalismo industrial en Inglaterra en el siglo XIX, que coincidió con la moral victoriana, la división que asocia el trabajo productivo con la producción de mercancías y el reproductivo con la esfera doméstica ignora que el trabajo de reproducción social produce aquello que hace posible la explotación en el capitalismo, es decir, los seres humanos. La separación entre producción y reproducción también tiene un carácter gentrificado, ya que el trabajo de cuidado se considera un “deber” de las mujeres en las sociedades patriarcales. Este proceso no solo ha naturalizado la responsabilidad de las mujeres en el cuidado, sino que ha institucionalizado nuevas formas de subordinación: la explotación, el despojo y la domesticación del trabajo, envueltos en la ideología del “amor”^(5,6)^.

En este sentido, la explotación se refiere a la extracción de plusvalía del trabajo asalariado, en la que el trabajador “doblemente libre” recibe un salario equivalente al costo de su reproducción (es decir, solo lo suficiente para mantenerse con vida, en condiciones de explotación continua), mientras que el capital se apropia del valor excedente producido. Se le considera “doblemente libre” porque puede vender su fuerza de trabajo, pero también porque no dispone de los medios de producción, enredándose contradictoriamente en las relaciones de intercambio. En otras palabras, el proceso ocurre bajo la apariencia de un libre intercambio de equivalentes, pero implica la apropiación sistemática del tiempo de trabajo excedente por parte del capital. La expoliación, por su parte, consiste en la captura directa y coercitiva de recursos, tierras y mano de obra que hacen posible la expansión del capitalismo, incluida la colonización de países periféricos, como Brasil. A diferencia de la explotación, que presupone un trabajador formalmente libre, la expoliación opera mediante violaciones, confiscaciones brutales y saqueos, a menudo asociados con procesos de racialización, colonialismo y subordinación política. Más aún, la expoliación no es un fenómeno en extinción ni se limita a los orígenes del capitalismo, sino que es una característica estructural que reduce los costos de producción para mantener la acumulación de capital a costa de poblaciones subyugadas, racializadas y migrantes. A diferencia del trabajo formalmente explotado, la expoliación no cubre los costos de vida de los hombres y mujeres racializados que conforman la mano de obra barata, subalterna y precaria^(6)^.

La tercera dimensión, la domesticación del trabajo, se refiere al conjunto de actividades de cuidado para el sustento de la vida que históricamente se han sentimentalizado como amor, inherentes a las mujeres y, por lo tanto, constituidas como trabajo invisible, desvalorizado y, con frecuencia, no remunerado. El término “domesticado” no se refiere al espacio doméstico, sino que expresa un proceso histórico de domesticación de los cuerpos y las mentes de las mujeres. Incluye también la naturalización de las actividades de cuidado, que pasan a percibirse como expresiones de afecto, una obligación moral o una vocación —y no como trabajo. Obviamente, las tres dimensiones (explotación, expoliación y domesticación) no actúan de manera aislada, sino que constituyen un sistema integrado de apropiación del trabajo y de la vida. Mientras que la explotación extrae valor del trabajo asalariado, la expoliación proporciona recursos y reduce costos, junto con la domesticación que disciplina los cuerpos, allanando el camino para la usurpación simbólica y material. Desde esta perspectiva, el capitalismo no se sustenta únicamente en la explotación económica formal, sino por la apropiación sistemática y domesticada del trabajo de reproducción social, visto como un recurso natural e infinito, aunque inscrito en la contradicción entre el capitalismo y el cuidado^(5,6)^.

Entender el cuidado como trabajo de reproducción social permite situarlo en el centro de la crisis del capitalismo, poniendo de manifiesto que no es un complemento de la extracción de plusvalía, sino su condición de posibilidad. El cuidado constituye, por lo tanto, no una dimensión ética, afectiva, “bondadosa” o relacional de la vida social (o de las mujeres), sino un elemento estructural de la economía política, cuya invisibilización es funcional a la acumulación capitalista. La perspectiva del cuidado como trabajo de reproducción social permite desentrañar la desvalorización del trabajo de las mujeres en la enfermería, ya que las condiciones históricas dependen de la forma en que nos insertamos, confrontamos o reeditamos el orden social capitalista que “canibaliza” el cuidado. En este sentido, hemos visto que la producción científica de la enfermería, en general, ensalza una de las principales formas de domesticación del cuidado en el capitalismo, disfrazándolo como “amor”, con refinamientos de apelaciones morales y toques del discurso de autoridad de la ciencia^(15)^. En contrapartida, en el siguiente tema descuidamos, confrontamos y desmoronamos la concepción de “amor” que viola a las mujeres en la enfermería.

## DESCUIDAR, CONFRONTAR Y ESTREMECER EL “AMOR” EN LA PROFESIÓN

Analicemos qué significa realmente “amor” en el discurso propio de la profesión y por qué se confunde con el cuidado. ¿Qué características asume el amor, qué dice o qué oculta? ¿De dónde surge la premisa de que el amor es siempre “bondadoso”, al igual que el cuidado y las enfermeras que lo prestan? ¿Qué significa realmente “bueno” y quién nos impone la bondad a nosotras, las mujeres? ¿Por qué deseamos parecer buenas, caritativas, altruistas, heroínas y santificadas, si somos humanas, convertidas en “Mujeres”^(23)^, portadoras de un cuerpo que desea y sufre, perecederas e imperfectas? ¿Por qué el discurso del “cuidado como amor”, explícitamente idealizado, atrae a parte de las mujeres de la enfermería? ¿De qué nos escondemos y por qué resulta tan seductoramente reconfortante para nuestros oídos? Muchas de estas preguntas no encajan en el alcance de este artículo, pero las planteamos como un ejercicio de reflexión descuidada. Quedémonos, entonces, con lo principal: lo que se dice sobre el “amor” y sus significados en la enfermería.

Al igual que ocurre con las indefiniciones sobre el cuidado, el amor no aparece como objeto de reflexión o profundización teórica en la ciencia de la enfermería, sino que se alude a él como una “bondad” autoevidente, en la que “el amor y el cuidado son dos datos universales”^(24)^, de carácter dogmático e inmune a la crítica. Los términos “cuidado”, “amor” y “enfermería” son intercambiables, tratados como sinónimos de atributos meramente morales, asumidos como una “ideología del cuidado”, “un ideal y un valor del cuidado […] que tendrá que convertirse en un deseo”. Algunas de las imprecisiones conceptuales quedan patentes en la expresión “Cáritas” (de origen léxico “caridad”), presentada como “una conexión explícita entre el cuidado y el amor”^(4)^; o incluso como “atemporal”; “núcleo de la ciencia y de la enfermería”; “las fuerzas cósmicas más asombrosas y misteriosas”; “la esencia de la enfermería y el eje más central y unificador de la práctica”. El cuidado, la enfermería y el amor se entrelazan de tal manera, añaden, que “tenemos que imponer a nuestro comportamiento, y no al de los demás, nuestra voluntad de cuidar y amar”^(24:90)^. En el mismo tono imperativo, los “Diez procesos Cáritas” sintetizan los mandamientos del cuidado para la enfermería, los cuales incluyen el “perdón”, los “valores altruistas “ de la bondad, la empatía, la preocupación y el amor; la aceptación y la “apertura al milagro”^(4,24)^, un vocabulario muy cercano al decálogo católico.

El discurso del amor-cuidado-enfermería genera ilusiones de perfeccionamiento, de divinización, de ascetismo y de “descorporificación como supuesta superioridad de las enfermeras y los enfermeros”, afirma una de las críticas más incisivas formuladas por un investigador del área^(25)^. De hecho, la autoexaltación hiperbólica de las enfermeras y los enfermeros corre el riesgo de caer en el desvarío. La práctica de la enfermería y quienes la ejercen se denominan “un misterio que hay que vivir”, que estaría “más allá de lo objetivable” y “preocupado por verdades ocultas”. La enfermería no es un trabajo situado históricamente en el tiempo y el espacio (y, por lo tanto, sujeto a presiones para cambiar), sino “un concepto filosófico que sugiere ternura” y “pasión”, en el que “se ofrece la promesa de preservar al ser humano en la sociedad”. Los enfermeros — nunca se utilizan formas de género en el lenguaje— son aquellos que “ven el mundo de manera diferente al paradigma de la ciencia médico-natural”, pues cuidan “de la totalidad de la personalidad humana”. Son seres espiritualmente evolucionados, “creativos que se autotranscenden”, quienes deben “tomar en cuenta algunas ideas metafísicas y morales como guías para sus propios esfuerzos”. De la enfermera se afirma además que sería la “única que escucha y honra la historia del otro y toda la intensidad de los sentimientos”, pues “puede tener acceso indirecto a la mente de una persona y a su interior”, por lo que estaría “capaz de formar una unión con otra persona, en un nivel que trasciende lo físico”^(4,24)^.

Los lectores y lectoras estarán de acuerdo con nosotros en que se trata de una visión de la enfermería completamente desconectada de la realidad del trabajo de cuidado, además de políticamente nociva para enfrentar las desigualdades sociales en la enfermería^(10,11,12,13,14,15,16,17,18,19,20)^. Decir que se trata de un “ideal”, notoriamente abstracto, no exime a las defensoras del “amor-cuidado” de las consecuencias opresivas sobre la realidad concreta de las enfermeras y técnicas, la mayoría de quienes se ajustan al mito del amor materno/romántico^(5,6,21,23)^. Que la teoría y los procesos caritativos del cuidado sean inaplicables en la práctica profesional es una obviedad^(2,3,25)^, dado el flagrante grado de fantasía de las concepciones transcritas. Las posiciones críticas de esta corriente atestiguan que el cuidado-amor es “solo un ideal entre otros”, políticamente poco realista, que ignora y fortalece las poderosas estructuras de poder en nuestra contra. Las reflexiones continúan al afirmar que este paradigma del cuidado fue concebido para ser “inefable”, permanentemente alusivo, deseoso de ejercer su poder sobre los demás e inflar la autoestima de la profesión^(25)^.

En consonancia con estas contraposiciones, destacamos la perspectiva del cuidado como trabajo de reproducción social, entendida aquí como expresión del descuido crítico-discursivo de las falacias del amor mitificado en las relaciones capitalistas. Asumimos la interpretación feminista del amor como una ideología mitificada que viola simbólicamente a las mujeres y fortalece la dominación masculina^(5,6,21)^. Bajo el patriarcado, el amor como inversión libidinal está estructurado para domesticar los cuerpos femeninos, moldeándolos para que permanezcan subyugados como “segundo sexo” (objeto), mientras que el “primero” pertenece al universo masculino (sujeto)^(23)^. Desde esta perspectiva, ante la disparidad entre el pretencioso “amor-cuidado” y las condiciones de trabajo indignas de las enfermeras y técnicas que prestan asistencia, cabría preguntarse cómo los ideales de pureza y perfección se han interiorizado tan profundamente en la profesión. En la contextualización sociohistórica del “capitalismo caníbal” realizada por Nancy Fraser, encontramos una pista importante. Hay tres hechos históricos en la consolidación del capitalismo en el siglo XIX que se entrelazan de manera nefasta en la profesionalización de la enfermería moderna, a saber: i – el fortalecimiento del capitalismo industrial y de Inglaterra como centro de poder político y económico; ii – la moralidad de la era victoriana; iii – el movimiento feminista inglés contra la Ley de Enfermedades Contagiosas. Explicaremos con más detalle cómo estos hilos se entrelazan en la enfermería profesional.

Comenzando por el primer punto, el fortalecimiento del capitalismo en la era industrial ratifica la separación entre el trabajo productivo (económico, masculino) y el reproductivo (social, femenino), construida gracias a la colonización y domesticación de las mujeres como “amas de casa” y “ángeles del hogar”. En este sentido, el esfuerzo por garantizar la reproducción social restringida al trabajo no remunerado se entrelazó vigorosamente con la dominación masculina. En Inglaterra —y este es el segundo punto—, el capitalismo y la dominación masculina coinciden con el puritanismo de la era victoriana y el surgimiento del modelo de enfermería de Nightingale, cuyo poder se extiende por todo el mundo. En este contexto de austeridad moral, el arraigo del capitalismo industrial se dio de manera diferente para las mujeres pobres en comparación con las ricas, con insatisfacción en ambos lados. Las mujeres pobres racializadas no cumplían con los ideales victorianos de domesticidad (recato, sumisión al esposo o al padre, restricción a las tareas domésticas o actividades consideradas femeninas, regulación del cuerpo y del comportamiento social). Por otro lado, las mujeres ricas, como Florence Nightingale, disfrutaban de comodidad material y prestigio moral, pero clamaban por libertad y autonomía jurídica. Según Nancy Fraser, la “solución” de la separación entre el trabajo productivo y el reproductivo se dio a costa de las mujeres, enfrentándolas unas contra otras. El conflicto consistía en lo siguiente: las preocupaciones filantrópicas de las mujeres ricas y de clase media de la época victoriana divergían de los intereses materiales de las “compañeras caídas en desgracia”, acentuadas por juicios morales respecto al ejercicio de la prostitución^(5)^.

El punto álgido del enfrentamiento moral entre las mujeres en la era victoriana culminó con la lucha por la derogación de la Ley de Enfermedades Contagiosas (*Contagious Diseases Acts*), encabezada por el movimiento “Revuelta de las Mujeres” (1864 a 1869), tercer punto en la historia de la enfermería^(26)^. Dicha ley tenía como objetivo controlar las infecciones de transmisión sexual entre los militares y, para ello, sometía a las mujeres prostitutas a exámenes ginecológicos forzados y a internamiento obligatorio. Curiosamente, aunque por razones opuestas, esa ley indignante unió a mujeres de distintas clases sociales, lideradas por el movimiento feminista de la Asociación Nacional de Damas (*Ladies National Association*), que denunciaba las leyes como discriminatorias y violentas. Las trabajadoras sexuales, las principales afectadas, se rebelaron igualmente contra las agresiones sufridas. Algunas damas de la aristocracia se unieron al movimiento por razones filantrópicas; otras, como Florence Nightingale, por razones morales, con el argumento de que la ley “sancionaba oficialmente el vicio” de la prostitución. Aunque la ley fue derogada, las repercusiones de ese acontecimiento reafirmaron las normas de pureza social y otorgaron nuevos poderes policiales de vigilancia sobre la clase obrera^(26)^.

Pero, ¿cómo es que la profesionalización de la enfermería moderna se ve influenciada por contextos históricos aparentemente tan distintos? A nuestro ver, el auge del capitalismo y el poder de la clase aristocrática, el puritanismo y los valores de blanqueamiento y pureza social que de él derivan, se incorporaron al modelo nightingaliano, en el que supuestamente se atribuye a las *lady nurses* un estatus de superioridad moral frente a las enfermeras técnicas, con la subyugación de las mujeres y la vigilancia de los cuerpos. Que este modelo moral de enfermera estándar influyó en la formación en todo el mundo, incluso en países colonizados, racializados y brutalmente expoliados como Brasil, es ampliamente discutido en la literatura^(7,8,9)^. El enfoque moralizante y cientificista del modelo nightingaleano no logró mitigar las contradicciones de las mujeres reales, con cuerpo, raza y clase social, lo que dejó como resultado injusticias y opresiones en el trabajo, tanto fuera como dentro del equipo de enfermería^(10,11,12,13)^. Sin embargo, la disciplina impuesta a las mujeres —que constituyen el 80 % de la fuerza laboral en el sector de la salud— requirió un ingrediente adicional, el más poderoso, encarnado en el apodo del “ángel blanco”, el rostro mítico de la “enfermería-cuidado-amor”. Ahora bien, la falaz candidez abnegada de la enfermería no es más que la colonización de las mujeres por la ideología del “ángel del hogar”, trasladada al hospital, lo que naturaliza la separación entre producción y reproducción capitalistas^(5,7,8)^.

Ante la historicidad descrita, observamos que la noción de “cuidado-amor” aún prevalece en el discurso científico, en la formación y en la práctica de la enfermería, lo que da lugar a fragilidad política y desigualdades. Una de las evidencias del autoritarismo moral de la profesión se manifiesta en la afirmación de que “tenemos que imponer a nuestro comportamiento […] nuestra voluntad de cuidar y amar”^(24:62)^, retrato fiel de la domesticación del trabajo de enfermería^(5,6)^. No es raro que el cuidado de enfermería se ensalce como “obligación o deber”, cuando en realidad se trata de la expoliación del trabajo emocional, entendido como el esfuerzo por gestionar, suprimir o expresar emociones específicas exigidas en el desempeño de una función, con el fin de cumplir con las expectativas normativas. El trabajo emocional constituye una de las formas de opresión del trabajo de reproducción social^(27)^. Además de las explotaciones emocionales en el trabajo formal, la exigencia del “cuidado-amor” expresa el grado de rigidez moral endógena a la enfermería. Al respecto, las investigaciones señalan la alta prevalencia del síndrome de *burnout* en enfermeras y técnicas, así como el agotamiento derivado de los trabajos emocionales de la profesión. Sin embargo, en general, el trabajo emocional y de reproducción social no son cuestionados por las investigadoras en enfermería, sino que se consideran atributos de la profesión, lo que renueva la fuerza opresiva del “amor”^(11,28)^.

Una vez que se han descuidado discursivamente las falacias del “amor” en la enfermería, que contrastan con la dura realidad laboral de la profesión, comparemos el autodiscurso con sus bases teóricas. Cuestionemos primero la concepción “metafísica” inespecífica y controvertida del cuidado, descrita a lo sumo como una dimensión espiritual que estaría “fuera del tiempo”^(1,4,24)^. Se trata de una simplificación burda, supuestamente basada en la noción de *Dasein* (aquel que posee la capacidad de cuestionar el sentido del ser) de Heidegger. Por limitaciones de espacio y propósito, no cabe desarrollar todo el debate en torno a conceptos filosóficos profundos; estaríamos actuando con el mismo grado de superficialidad que cuestionamos. Sin embargo, nos permitimos delimitar el centro de la controversia.

La metafísica es una tradición filosófica dedicada a la búsqueda de los universales de la verdad, que ha pasado por diversos giros y críticas, siendo la principal la realizada por Heidegger. Su cuestionamiento consiste en el olvido, por parte de la metafísica tradicional, de la pregunta por el ser (algo que subsiste por su sentido, inaprensible mediante un concepto), para pasar a ocuparse de los entes (lo que decimos, lo que entendemos, interpretamos; es lo que es, como somos). En la visión heideggeriana, al indagar por el “ser” del ente, a través de la noción de *Dasein* (ser humano) que se abre a esa revelación, la filosofía superaría a la metafísica clásica, lo que supone una intervención crítica, al tiempo que “destrucción” de la tradición, especialmente por la innovadora comprensión de la dimensión temporal del ser. Así, la afirmación de que el cuidado sería “atemporal” contradice frontalmente la base fundacional del *Dasein*, pues el ser solo existe en el tiempo (de ahí el nombre “Ser y tiempo”)*,* además de situar a la enfermería fuera de la historia, incapaz de cambiar su destino^(29)^.

La segunda contradicción se refiere a la supuesta representación “de la herencia y los fundamentos de Nightingale”^(4)^en la noción sentimental del cuidado como “bondad”, “amor”, “ternura”, “compasión” y conceptos relacionados. A pesar del puritanismo moral y el protestantismo que tanto influyeron en el conservadurismo de la profesión, sabemos muy bien que Florence Nightingale defendía una ciencia racional y empírica para el cuidado de enfermería, mostrándose reacia a los sentimentalismos fáciles. De este modo, algunas de sus posturas contradicen frontalmente la noción sentimental atribuida al cuidado-amor, por ejemplo: “No es tarea de la enfermería ser bondadosa, en el sentido de hacer lo que uno quiera o satisfacer los propios sentimientos; su tarea es cumplir con las leyes de la salud”; o también: “una enfermera no es una mujer devota y obediente, sino que debe ser una persona reflexiva e instruida”^(30:200)^. Con esto, la supuesta representación de los pensamientos de Florence en la noción del cuidado como amor se desmorona al contradecir sus cimientos.

Una vez que descuidamos y cuestionamos el discurso sobre el amor, llegamos al punto de ruptura, el estremecer, momento en el que nos permitimos abstracciones, incomodidades, cuestionamientos, críticas y autocríticas. Retomemos las preguntas iniciales, especialmente el motivo por el cual el discurso idealizado del “amor” atrae a parte de las mujeres de la enfermería, o por qué resulta tan seductoramente reconfortante para nuestros oídos. Quizás el dolor recóndito que acariciamos con autoengaños sea el mismo que nos une en la condición de “Mujer”, como el “Otro” del “Yo” universal, relegada a la condición de objeto designado por la autoridad del sujeto masculino. En el libro II de *El segundo sexo*, Simone de Beauvoir analiza las experiencias amorosas de la mujer, lo que hace con su condición, cómo se siente y qué evasiones se le permiten. A partir de los entrelazamientos de las categorías de la “narcisista” (el amor a uno mismo es un fin absoluto), la “apasionada” (mitificación del amor romántico) y la “mística” (entrega y devoción del amor a Dios), la filósofa reflexiona sobre las circunstancias que llevan a las mujeres a volverse hacia sí mismas y dedicar su amor, como una especie de compensación por lo que se les había negado como sujeto^(23)^.

En consonancia con las reflexiones de Beauvoir, parece que la necesidad de autoafirmación, superioridad moral e identidad inmutable expresada en los discursos de la enfermería se entrecruza con el dolor latente del “Otro” que nos pertenece a todas, mujeres condicionadas por el tiempo y por lo que hacemos en él. Además, “esos esfuerzos de salvación solo pueden resultar en fracasos; o bien la mujer se relaciona con algo irreal —su doble o Dios—; o bien crea una relación irreal con un ser real”^(23:876)^.

## CONSIDERACIONES FINALES

Necesitamos hablar sobre el cuidado como trabajo de reproducción social en la enfermería, incluir la perspectiva de género y el diálogo fructífero con la epistemología feminista en los planes de estudio de pregrado y posgrado, con repercusiones en las producciones científicas y en la práctica política de la profesión. En lugar de la búsqueda improbable de “universales” de la verdad o de misterios exotéricos que no contribuyen a la praxis ni al perfil crítico registrado en los proyectos pedagógicos de los cursos, ampliemos la visibilidad de la resistencia crítica y autocrítica de la producción científica de las enfermeras investigadoras. Con ello, desvelamos los discursos totalitarios del “amor” que aprisionan nuestro actuar reflexivo y nuestra capacidad de construir la historia de la profesión. La ciencia que producimos debe cuestionar las contradicciones sociales y de género en la enfermería, cuyo trabajo ha sido rigurosamente domesticado en los programas de formación de enfermeras y técnicas de enfermería. Cuestionemos igualmente la supuesta neutralidad científica entretejida con la búsqueda egocéntrica de evidencia como discurso de autoridad, desvelando su compromiso ideológico con el orden social capitalista machista, misógino, sexista, racista y antiecológico que nos devora bajo falsas cantinelas de nobleza. El conocimiento que producimos no puede eximirse de la dimensión política ni del compromiso con la mejora de las condiciones laborales, la revalorización salarial y la lucha contra las formas de violencia en la profesión, entre otras agendas inaplazables.

Para trazar una ruta crítica del cuidado en la enfermería, reconozcamos, por fin, que nuestra riqueza consiste precisamente en lo que ha sido sistemáticamente borrado por el conservadurismo y el elitismo de la enfermería moderna: la diversidad de culturas, géneros, sexualidades, razas, clases y saberes de las mujeres que trabajan arduamente por la salud de la población brasileña, en los distintos espacios de la reproducción social, no solo en la atención. Quizás con esto podamos inaugurar otras formas de amor, más cercanas a la dimensión política y económica acorde con la democracia de las mujeres.

## Data Availability

Todo el conjunto de datos que apoya los resultados de este estudio fue publicado en el propio artículo.
